# Direct observation of the molecular mechanism underlying protein polymerization

**DOI:** 10.1126/sciadv.abm7935

**Published:** 2022-08-31

**Authors:** Nikolas Hundt, Daniel Cole, Max F. Hantke, Jack J. Miller, Weston B. Struwe, Philipp Kukura

**Affiliations:** ^1^ Physical and Theoretical Chemistry Laboratory, Department of Chemistry, University of Oxford, South Parks Road, Oxford OX1 3QZ, UK.; ^2^ The Kavli Institute for Nanoscience Discovery, Oxford, UK.; ^3^ Department of Physics, Clarendon Laboratory, University of Oxford, Parks Road, Oxford OX1 3PT, UK.; ^4^ The PET Research Centre and The MR Research Centre, Aarhus University, Aarhus, Denmark.

## Abstract

Protein assembly is a main route to generating complexity in living systems. Revealing the relevant molecular details is challenging because of the intrinsic heterogeneity of species ranging from few to hundreds of molecules. Here, we use mass photometry to quantify and monitor the full range of actin oligomers during polymerization with single-molecule sensitivity. We find that traditional nucleation-based models cannot account for the observed distributions of actin oligomers. Instead, the key step of filament formation is a slow transition between distinct states of an actin filament mediated by cation exchange or ATP hydrolysis. The resulting model reproduces important aspects of actin polymerization, such as the critical concentration for filament formation and bulk growth behavior. Our results revise the mechanism of actin nucleation, shed light on the role and function of actin-associated proteins, and introduce a general and quantitative means to studying protein assembly at the molecular level.

## Teaser

The mechanism of actin polymerization is revealed by quantifying the full range of intermediates during filament formation.

## INTRODUCTION

The formation of noncovalent polymers by proteins underpins a wide range of physiological processes such as cell division and migration, control of cell shape, and intracellular transport (*1*–*7*). At the same time, pathological conditions such as neurodegenerative disorders (*8*–*10*), type 2 diabetes (*9*–*11*), and cancer metastasis (*12*, *13*) are associated with undesired protein self-assembly. Direct access to the associated molecular mechanisms is thus critical not only for our understanding of physiological function but also of changes associated with disease, and for the development and optimization of routes to intervention. The formation of extended structures depends principally on molecular interactions, structure, and symmetry, resulting in a delicate balance between substrate availability, binding, and unbinding rates (*14*, *15*). In its simplest form, linear polymerization of proteins has been described by a nucleation and growth process, analogous to the condensation mechanism associated with gas-liquid phase transitions, where a kinetic barrier needs to be overcome by the gas to condense into a liquid (*16*, *17*). This behavior has been observed for many different biopolymers such as actin (*16*, *17*), microtubules (*18*, *19*), amyloid-β (*20*), α-synuclein (*21*), huntingtin (*22*), and islet amyloid polypeptide (*11*), establishing nucleation-based growth as the core principle of protein self-assembly (*23*).

Experimentally, the measured bulk signal of a solution of growing biopolymers exhibits a sigmoidal growth profile with a lag phase, representing the nucleation period at the onset of polymerization, a steep growth phase, and a plateau phase, as the amount of free polymer building blocks becomes limited. The underlying molecular mechanism is then extracted by finding kinetic models that reproduce the experimental data under different conditions and concentrations. The intrinsic challenge to this approach is that numerous different, at times contradictory, nucleation models provide satisfactory agreement between experiment and theory [reviewed in (*19*, *24*, *25*)]. Resolving these ambiguities could in principle be achieved by the direct observation and quantification of individual species ranging from monomers to complexes consisting of hundreds of oligomers representing the full range from globular protein to filament. This quantification, however, places considerable demands on resolution and dynamic range of any method given the heterogeneity of species in solution during polymerization.

**Fig. 1.**
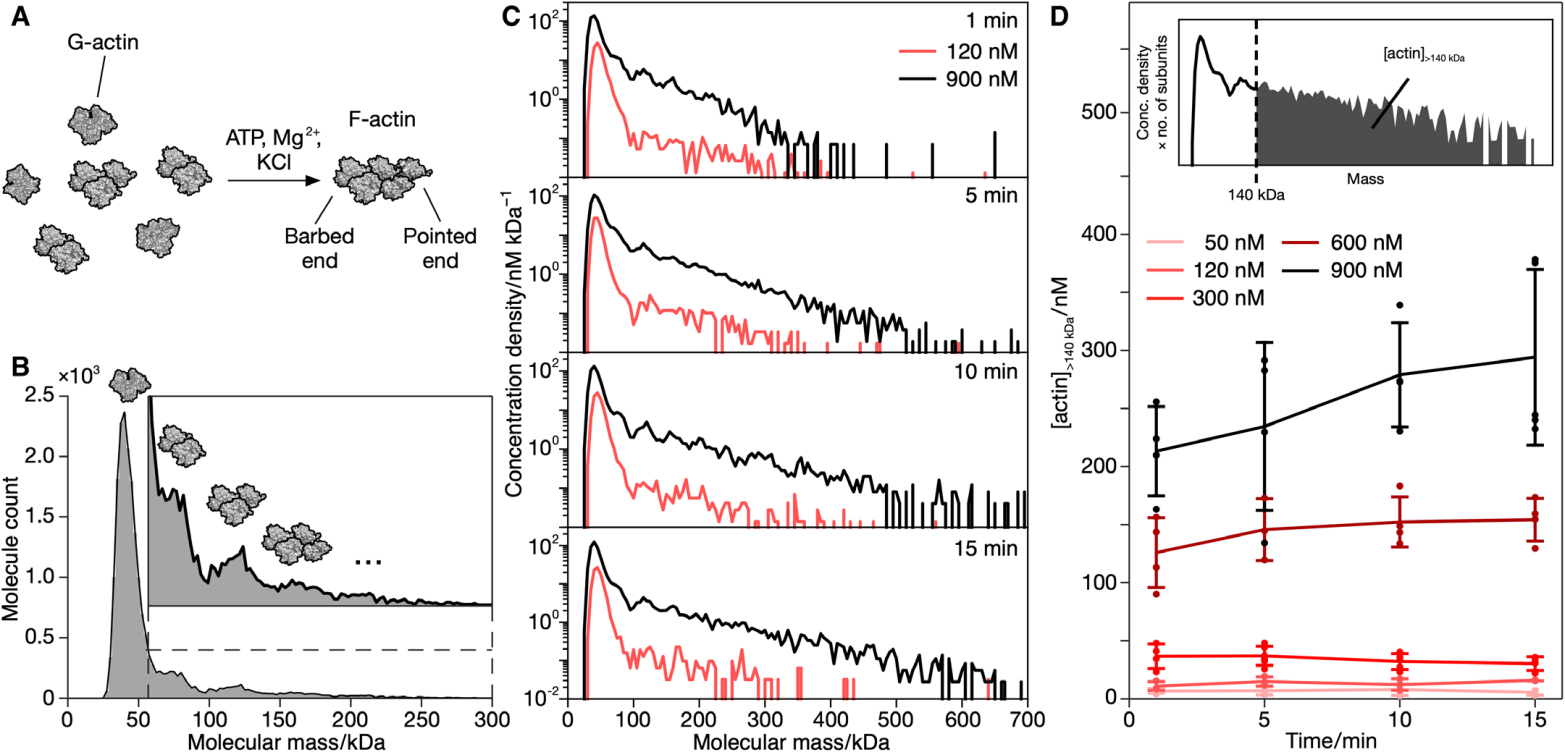
MP of actin filament formation. (**A**) Schematic of the transition from globular (G-) to filamentous (F-) actin, which is induced in vitro by the presence of ATP, Mg^2+^ ions, and KCl. The two distinct ends of the bipolar actin filament are indicated. (**B**) MP mass distribution of SEC-purified G-actin (*n* = 20,573 particles from four technical replicates). Inset: Magnification of the higher molecular mass peaks. (**C**) Evolution of oligomeric distributions at (120 nM) and above (900 nM) the critical actin concentration for filament formation as a function of time after inducing polymerization by adding 100 mM KCl and 2 mM MgCl_2_. Distributions are pooled from three to five technical replicates. Particle numbers: 120 nM: *n*_1min_ = 7348, *n*_5min_ = 5797, *n*_10min_ = 7017, *n*_15min_ = 2973; 900 nM: *n*_1min_ = 9000, *n*_5min_ = 29,314, *n*_10min_ = 14,211, *n*_15min_ = 19,668. (**D**) Time-dependent formation of actin species larger than 140 kDa for different actin concentrations. Corresponding mass distributions are shown in fig. S5. Circles represent technical replicates. Error bars indicate their SD. Inset: Mass distribution of 900 nM actin 15 min after inducing polymerization with the concentration density scaled by the corresponding number of actin subunits. The shaded area is used to quantify the total concentration of actin in species larger than 140 kDa.

We recently introduced mass photometry (MP), the label-free detection, and mass measurement of individual biomolecules and their complexes in solution (*26*). The ability of MP to resolve different oligomeric states, wide mass range of operation (40 kDa to 10 MDa), accurate quantification of relative object concentrations by molecular counting (*27*, *28*), and in principle unlimited dynamic range owing to label-free single-molecule detection are ideally suited to address the challenges associated with studying protein self-assembly. Here, we apply MP to study the early assembly steps of actin (Fig. 1A and fig. S1), due to its seminal role in our understanding of biopolymer formation, its extensive biophysical and structural characterization to date, and the number of different, at times contradictory, models that have been proposed for the molecular mechanism resulting in filament formation (Fig. 1A) (*29*–*38*).

## RESULTS

For purified actin, the transition from globular actin (G-actin) subunits to filamentous actin (F-actin) can be induced in vitro by changing its solution environment from low ionic strength with Ca^2+^ ions and adenosine triphosphate (ATP) to high ionic strength with Mg^2+^ ions and ATP, respectively (Fig. 1A, see Materials and Methods for detail) (*17*, *39*). As a first experiment, we investigated purified G-actin with MP. We found that G-actin, exhibiting a monodisperse profile in size exclusion chromatography (SEC; fig. S4A), actually contained small amounts of low-order oligomers as revealed by the high dynamic range achievable with MP (Fig. 1B). The distribution of these oligomers varied among different SEC fractions (fig. S4B). The high mass resolution of MP made it possible to differentiate small actin oligomers as separate peaks, enabling their individual quantification. At the critical actin concentration for filament formation (120 nM) (*40*), we found little changes in the oligomeric distribution after inducing polymerization (Fig. 1, C and D, pink). Repeating the same experiment at 900 nM actin resulted in a clear increase in the abundance of species with mass greater than 140 kDa, as expected for filament growth (Fig. 1, C and D, black). For comparison, no such shift in the oligomeric distribution was observed for G-actin, if the polymerization factors were not added (fig. S7).

**Fig. 2.**
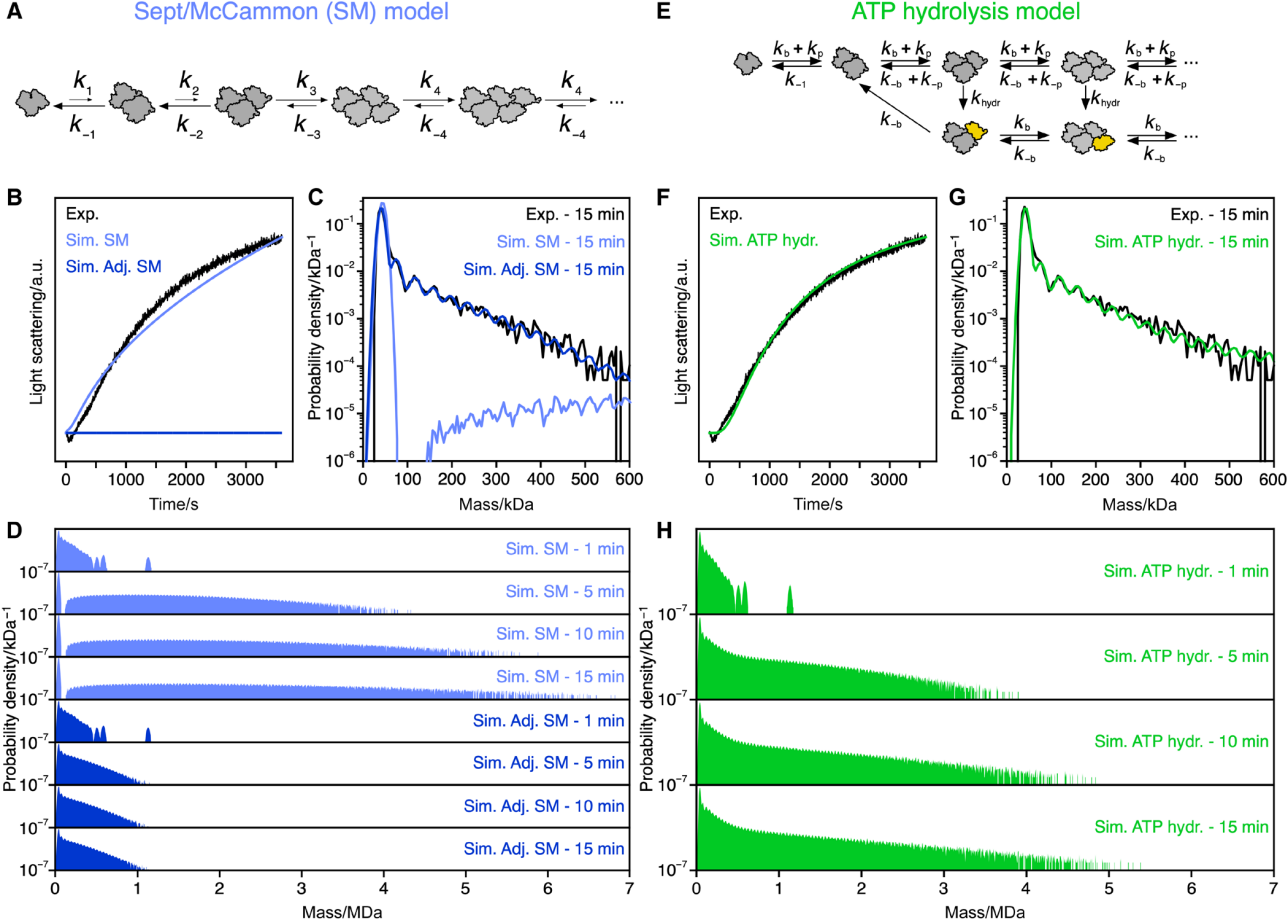
Comparison of kinetic models for actin polymerization. (**A**) Kinetic scheme summarizing the Sept/McCammon (SM) model. (**B**) Comparison of an experimental light scattering trace of 2 μM polymerizing actin (black, one of three technical replicates) with simulated scattering time courses for the same actin concentration based on the SM kinetic model with original rate constants (light blue, table S1A) and with adjusted rate constants (dark blue, table S1B). (**C**) Magnification of the low mass regime of the 15-min distributions in (D) and comparison with their corresponding experimental distribution determined with MP. (**D**) Time course of simulated mass distributions for 900 nM polymerizing actin based on the SM kinetic model with original rate constants (light blue, table S1A) and with adjusted rate constants (dark blue, table S1B). (**E**) Scheme summarizing a kinetic model where ATP hydrolysis stabilizes actin filament pointed ends (golden subunit). (**F**) Comparison of an experimental light scattering trace of 2 μM polymerizing actin (black) with a simulated scattering time course for the same actin concentration based on the kinetic model in (E) (green). (**G**) Magnification of the low mass regime of the 15-min distribution in (H) and comparison with its corresponding experimental distribution determined with MP. (**H**) Time course of simulated mass distributions for 900 nM polymerizing actin based on the kinetic model in (E) (green).

On the basis of these results, we decided to test how the experimental mass distributions compared to those predicted by existing nucleation-based models for actin filament formation. To enable quantitative comparison of kinetic models with our experimental results, we simulated the evolution of the oligomeric distribution with time, based on the underlying kinetic model. As an additional, more traditional experimental dataset, we recorded the temporal evolution of bulk light scattering (*29*) and compared it with our simulations (see Materials and Methods). We started with a representative nucleation-based model (Sept/McCammon) (*35*) relying on free energy calculations to determine theoretical kinetic rate constants for the conversion between all possible actin oligomers, which concluded that the tetramer is the smallest stable oligomer with association and dissociation rates equal to any larger species (Fig. 2A and table S1A).

We found good agreement when comparing simulated with experimental bulk scattering traces (Fig. 2B, light blue), as in the original report (*35*). The mass distributions exhibited filament growth, reaching species containing hundreds of actin subunits in 15 min (Fig. 2D, light blue). Close inspection of the low mass regime (<600 kDa), however, revealed considerable discrepancies between simulation and experiment (Fig. 2C). While the nucleation-based model predicts low or negligible steady-state levels of small oligomers as expected for nuclei that are unstable compared to both smaller and larger species (Fig. 2C, light blue), MP detected substantial amounts of small actin oligomers at all probed time points after the onset of polymerization (Figs. 1C and 2C, black). In a first attempt to compensate for these discrepancies, we adjusted the rate constants in the nucleation-based reaction scheme (adjusted Sept/McCammon model; table S1B) until our simulation agreed well with the MP data at low mass (Fig. 2C, dark blue). This rate constant set, however, resulted in a complete lack of long filament growth evidenced in both the simulated mass distribution of oligomeric species (Fig. 2D, dark blue) and the simulation of bulk scattering (Fig. 2B, dark blue).

The substantial, and concentration-dependent, steady-state levels of early actin assembly intermediates during polymerization argue against a model in which the formation of small, kinetically labile, actin nuclei is the rate-limiting step during filament growth. The simple reaction scheme in Fig. 2A where the on- and off-rates (*k*_4_, *k*_−4_) are the same for any filament length does not support net growth of long filaments out of the pool of small oligomers found with MP. Instead, actin filaments stay smaller than 1 MDa (Fig. 2D, dark blue). We reasoned that there must be a different event that leads to the nucleated growth type behavior observed in bulk polymerization experiments. We therefore extended the reaction scheme by introducing the possibility for actin oligomers to transition from a state with one set of rate constants to a state with a different set of rate constants (see Fig. 2E and Materials and Methods: “Simulation of actin assembly” section, kinetic scheme 2). Simulations with this reaction scheme revealed that a slow transition of oligomers from a dynamic state to a state more resilient to disassembly causes a subpopulation of the short oligomers to grow into longer filaments.

It is generally assumed that filaments in Mg-ATP containing buffer assemble predominantly from ATP-actin, with distinct rate constants for the two different filament ends (Fig. 1A), the barbed end (*k*_b_ and *k*_–b_) and the pointed end (*k*_p_ and *k*_–p_) (*40*). Incorporation of subunits into filaments triggers actin’s adenosine triphosphatase (ATPase) activity, causing the subunits to transition first into an adenosine diphosphate (ADP)–inorganic phosphate (P_i_) state and upon phosphate release to an ADP state (*41*, *42*). Since subunits are incorporated faster at the barbed end, actin filaments usually adopt a state with an ATP-actin cap at the barbed end followed by ADP-P_i_ subunits toward the pointed end, which eventually are converted to ADP subunits upon slow phosphate release (*40*–*42*). Notably, Fujiwara *et al.* (*43*) reported a >10-fold slowdown of the turnover rates for ADP-P_i_ subunits at the pointed end.

We thus tested an actin nucleotide state-based interpretation of our kinetic stabilization model (Fig. 2E), where stabilization is caused by the conversion of the terminal subunit at the pointed end into the ADP-P_i_ state (golden subunit, Fig. 2E) with a transition rate equal to the ATP hydrolysis rate of filament-incorporated subunits, *k*_hydr_ (table S2A) (*44*). Assuming the simplest case in which all assembly and disassembly rate constants are equal except for the disassembly of a dimer, which has fewer contact sites between subunits (*29*, *45*–*47*), enabled us to use independently determined rate constants for all steps except for *k*_−1_. Strikingly, a simulation using literature values for the filament end-specific rate constants *k*_b_, *k*_–b_, *k*_p_, and *k*_–p_ (*40*), the filament-internal ATP hydrolysis rate *k*_hydr_ (*44*), and a single, manually optimized value for dimer dissociation (*k*_−1_) reproduced both the MP data and bulk scattering trace (Fig. 2, F to H). For comparison, a reaction model without a stabilizing transition (fig. S11, A and C, purple) or restricting growth to the barbed end without a preceding transition (fig. S11, B and C, yellow) both failed to produce long actin filaments.

To further validate our ATP hydrolysis–dependent model, we compared the model predictions with MP data at different actin concentrations both below and above the critical concentration for filament formation (fig. S12). For all concentrations, we found excellent agreement between simulation and experiment. To further compare the two kinetic models tested using information theory–based metrics for model selection, we undertook Markov chain Monte Carlo (MCMC) sampling to comprehensively explore the parameter space and systematically find the best kinetic parameters for each of our models (*48*). Applying the MCMC approach to the Sept/McCammon model required variation of kinetic parameters by more than five orders of magnitude without finding a marginally preferential fit (figs. S13B and S14). By contrast, the nine-dimensional parameter space of our ATP hydrolysis model had to be adjusted only to a negligible degree, often less than 10% of literature rate constants (fig. S13A), to find an optimized fit (figs. S14 and S15), providing an information theory–based validation of our new model.

**Fig. 3.**
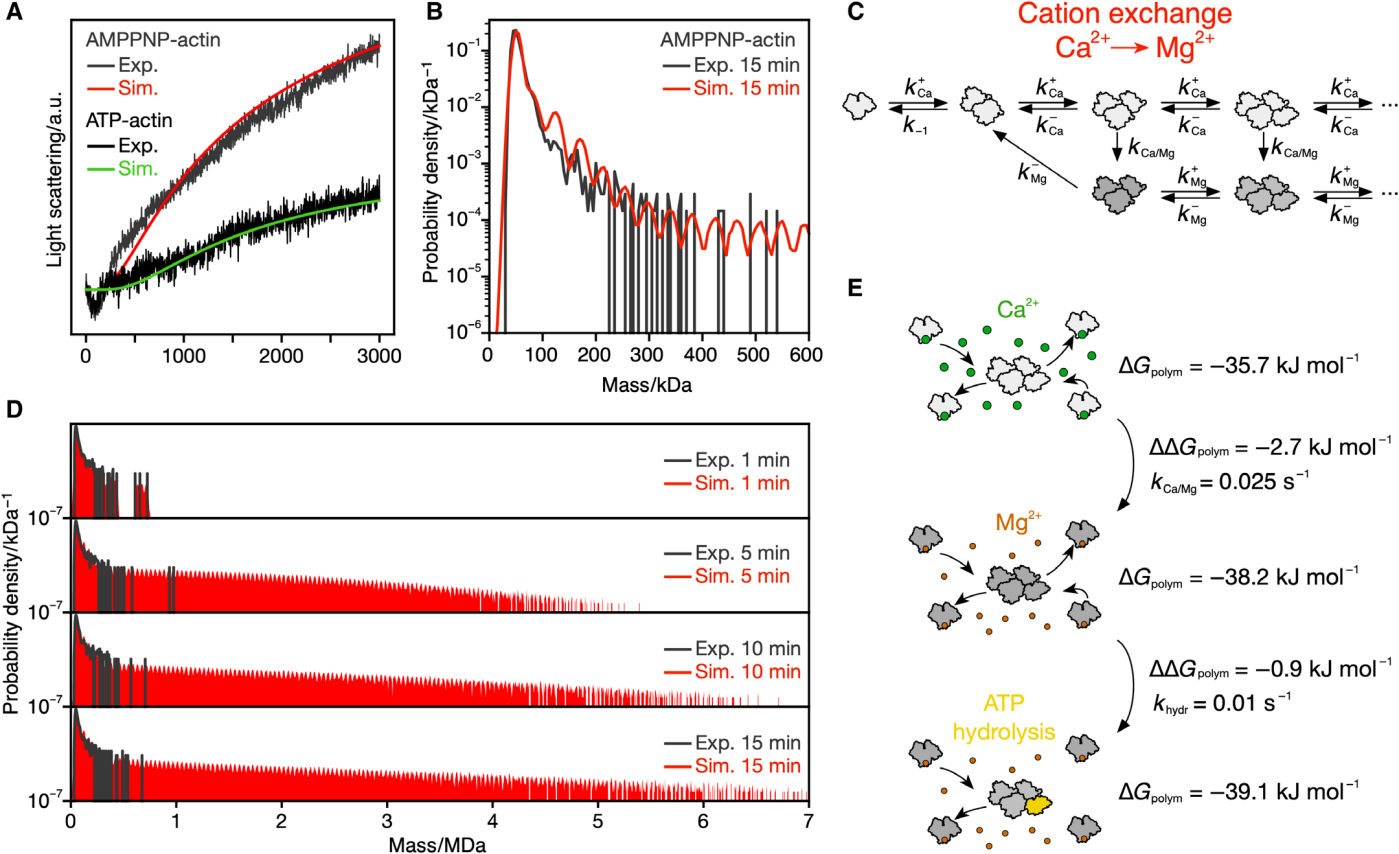
ATP hydrolysis–independent model for actin polymerization. (**A**) Experimental light scattering traces of 2 μM polymerizing AMP-PNP-actin (dark gray, average from four technical replicates) and ATP-actin (black, average from two technical replicates) and comparison with simulated light scattering traces based on the kinetic model in (C) (red) or Fig. 2E (green). (**B**) Magnification of the low mass regime of the 15-min distributions in (D). (**C**) Kinetic scheme summarizing a model where exchange of Ca^2+^ to Mg^2+^ on actin changes the polymerization rates. (**D**) Time course of simulated mass distributions for 900 nM polymerizing AMP-PNP-actin based on the kinetic model in (C) (red) and comparison with the corresponding experimental distributions (dark gray). (**E**) Summarizing model of actin polymerization. At least three different states of actin, i.e., with bound Ca^2+^ and ATP (top), Mg^2+^ and ATP (middle), and after ATP hydrolysis in the terminal subunit (bottom), are characterized by different Gibbs free energies of polymerization (Δ*G*_polym_; compare table S2). A slow transition from a state with less favorable Δ*G*_polym_ to one with more favorable Δ*G*_polym_ leads to the formation of long filaments. The difference of Δ*G*_polym_ between the states and the transition rate dictates the resulting length distribution and polymerization dynamics.

These results demonstrate that the formation of actin nuclei as a rate-limiting step during actin polymerization is not required for explaining nucleated growth type behavior in bulk experiments. Instead, the ATP hydrolysis–driven transition from a state where actin (dis)associates from two ends to a state where polymerization occurs predominantly from the barbed end provides an alternative kinetic model. However, a major shortcoming of this model is that it does not explain why actin is also able to polymerize in the presence of ADP (*43*, *49*) and nonhydrolysable ATP analogs (*50*, *51*) and even without any nucleotide at all (*52*). To challenge our hypothesis, we exchanged ATP in G-actin by the nonhydrolysable ATP analog adenylyl-imidodiphosphate (AMP-PNP) and assessed both the time course of its mass distribution and bulk light scattering in AMP-PNP–containing buffer under polymerizing conditions. AMP-PNP-actin (2 μM) was able to polymerize as evidenced by the increase in light scattering (Fig. 3A), confirming previous studies (*50*, *51*). Intriguingly, the increase in bulk light scattering upon polymerization with AMP-PNP was stronger than in the presence of ATP (Fig. 3A, gray versus black trace), suggesting accelerated formation of long filaments in the presence of AMP-PNP. In MP, AMP-PNP-actin exhibited a similar oligomeric distribution as ATP-actin, confirming that nucleus formation is not the rate-limiting step during filament formation. However, steady-state levels of actin oligomers were lower for AMP-PNP-actin than for ATP-actin (compare Figs. 2G and 3B), despite faster apparent polymerization with AMP-PNP in bulk light scattering.

It is widely accepted that actin has specific cation-binding sites on its surface: a high-affinity site modulating its polymerization properties and several low-affinity sites that affect filament stiffness (*53*, *54*). It has been suggested that the exchange of Ca^2+^ ions bound to the high-affinity site by Mg^2+^ induces a slow conformational change that activates actin monomers for polymerization (*31*, *55*–*57*). Hence, we tested an interpretation of our kinetic model where the top reaction pathway represents polymerization with the rates of Ca^2+^-bound actin, the transition rate equals the isomerization rate from Ca^2+^- to Mg^2+^-bound actin, both reported by (*31*), and the bottom reaction pathway represents polymerization with the rates of Mg^2+^-bound actin reported by (*40*) (Fig. 3C).

The simulation convincingly reproduced both the time course of mass distribution and light scattering of AMP-PNP-actin (Fig. 3, A, B, and D, red). This result suggests that actin polymerization may be explained by transitions between different kinds of equilibria. The kinetics of ATP-actin polymerization appear to be determined by the ATP hydrolysis–induced transition (Fig. 2E), whereas AMP-PNP-actin polymerization is mainly controlled by the kinetic transition between Ca^2+^- and Mg^2+^-bound states (Fig. 3C). When comparing the relative amplitudes of the simulated light scattering curves from ATP hydrolysis and cation exchange model, they could explain the discrepancies between the amplitudes of the AMP-PNP and ATP experiment (Fig. 3A). Irrespective of the rate differences between the two models, however, they share the same underlying kinetic principle: Filament growth is enabled by switching a subset of species toward a state more favorable for polymerization.

## DISCUSSION

Our results have important consequences for our understanding of the processes that drive actin polymerization. In the absence of nucleation, a simple equilibrium with rate constants that are the same for each step in the polymerization only results in the formation of a steady state with an approximately exponential distribution of oligomers and low abundance of long polymers (Fig. 2D, dark blue, and fig. S11). Thermodynamically, this type of state is characterized by equal Gibbs free energies Δ*G*_polym_ for each step in the polymerization. With our new kinetic model, we could demonstrate that a slow transition from a state with a less favorable to a more favorable Δ*G*_polym_ leads to growth of long actin filaments and nucleated growth–like behavior from a bulk perspective (Fig. 3E). In the cases studied here, this may be achieved by a switch from Ca^2+^- to Mg^2+^-bound actin or by ATP hydrolysis. It seems to be either the slowest transition rate or the smallest difference between polymerization Gibbs free energies (ΔΔ*G*_polym_) that dictates the overall kinetics and distribution of species (Fig. 3E).

**Fig. 4.**
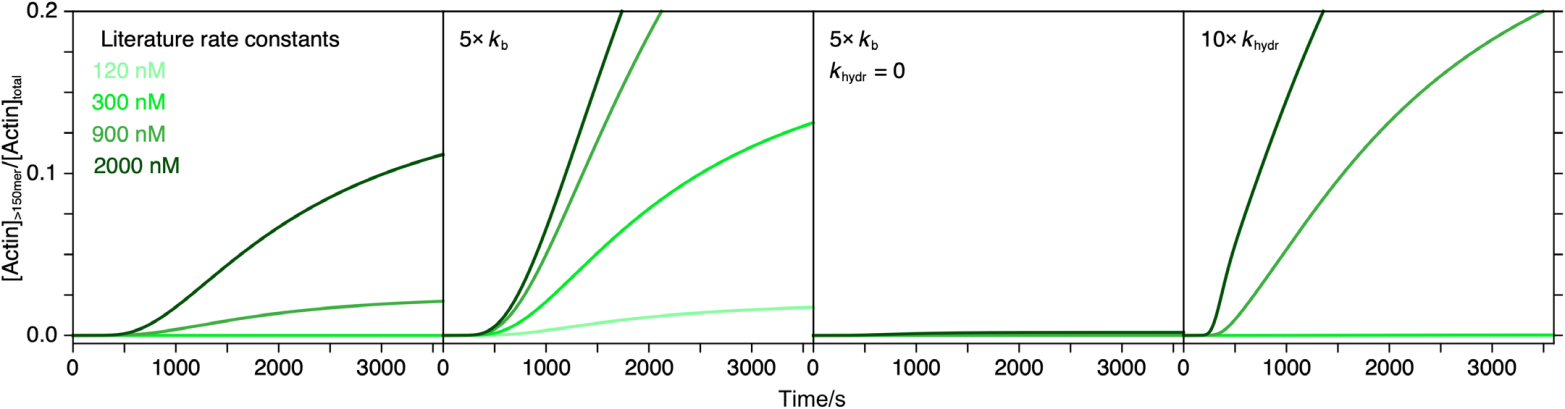
Implications of our kinetic model for actin binding proteins. Influence of barbed end elongation rate (*k*_b_) and ATP hydrolysis rate (*k*_hydr_) on filament formation (for filaments longer than 150 subunits). Light green to dark green correspond to 120, 300, 900, and 2000 nM total actin. First panel: Simulation based on ATP hydrolysis model with rate constants summarized in table S2A. Second panel: Same simulation with a fivefold increased value for *k*_b_. Third panel: Same simulation as second panel but with *k*_hydr_ = 0. Fourth panel: Same simulation as first panel but with 10-fold increased value for *k*_hydr_. For corresponding filament length distributions, see fig. S16.

As an illustration of how this new model may affect our understanding of the function of actin-binding proteins, we evaluated the effect of changing specific rate constants on filament formation, defined as the ratio of actin present in oligomers containing more than 150 monomers to the total actin concentration (Fig. 4 and fig. S16). In our first scenario, elevation of the elongation speed at the barbed end, represented here by a fivefold increase of the forward rate constant *k*_b_, accelerated production of longer filaments (Fig. 4, second panel, and fig. S16B). Under these conditions, filament formation proceeded even at 120 nM total actin concentration, implying a drop in the critical concentration. For actin binding proteins, this suggests that supporting subunit addition alone would be sufficient to serve as what has previously been considered a nucleating factor.

Actin nucleators, such as formins, are thought to operate based on capture and assembly of actin subunits into a stable nucleus (*58*, *59*). Since, according to our MP experiments, a steady-state pool of small actin oligomers is always available for elongation, a nucleation activity is not required. Instead, even for a fivefold accelerated barbed end growth rate (5× *k*_b_), a gradual stabilization of the pointed end mediated by ATP hydrolysis is crucial for net filament growth. In the absence of this kinetic transition (*k*_hydr_ = 0), filaments stay short, rarely growing beyond 150 subunits at 2 μM actin despite a faster barded end growth rate (Fig. 4, third panel, and fig. S16C), further highlighting that it is the transition between two distinct filament states, which drives net growth. Notably, the process of formin recruitment to actin itself may also represent a kinetic transition promoting long filament formation.

As an alternative scenario, we assessed the influence of an increased transition rate in our model by accelerating the transition rate at terminal actin subunits 10-fold (10× *k*_hydr_). Under this condition, filaments reached their maximum length quickly (Fig. 4, fourth panel) but remained short on average (fig. S16D), an effect similar to a rise in critical concentration.

These observations have important implications for interactors, which bind alongside or at the pointed end of an actin filament. These proteins may control both filament length and dynamics by regulating actin’s ATPase activity, cation binding, or pointed end stability. While many studies have investigated the influence of actin binding proteins on the dynamics of filament ends, little has been reported to date on their influence on ATP hydrolysis (*60*) and cation interactions.

Nucleated growth has been the predominant model to explain protein self-assembly (*11*, *17*, *18*, *20*, *21*, *29*, *30*, *35*). Despite the impressive technical advances in structural biology in the recent decades, characterization of protein polymer nucleation has been largely restricted to the bulk (*19*, *61*–*63*). Our experiments demonstrate that MP is capable of quantifying early intermediates of polymerization reactions, including those of very low abundance that nevertheless play a critical role in the process. It thereby helps to resolve the underlying molecular processes and facilitates interpretation of kinetic models. We anticipate that our approach, in combination with information from bulk and structural methods, will be transformative for our ability to investigate the mechanisms of protein self-assembly, with implications for our mechanistic understanding, routes to intervention (*64*), and the rational design of interactions where desired for novel function (*65*).

## MATERIALS AND METHODS

### Actin purification

Actin was purified on the basis of the procedures described in (*66*–*68*). The entire preparation was carried out at 4°C using degassed buffers unless otherwise stated. G-actin was extracted from 4 g of rabbit skeletal muscle acetone powder (Pel-Freez Biologicals) by stirring in 100 ml of G-actin buffer [2 mM tris-HCl (pH 8.0), 0.2 mM CaCl_2_, 0.2 mM ATP, and 2 mM dithiothreitol] for 30 to 60 min (fig. S3, fraction 1). Crude solid material was removed by filtering through a sieve. Residual insoluble material was removed by centrifugation at 30,000*g* for 1 hour (Beckman Optima L-90K ultracentrifuge using SW-32Ti rotor at 15,600 rpm) (fig. S3, fraction 2). The supernatant was supplemented with ^1^/_10_ volume of 10× KMEH (high salt) [1× concentrations: 10 mM Hepes-KOH (pH 7.5), 100 mM KCl, 2 mM MgCl_2_, and 1 mM EGTA] and incubated for 2 hours at room temperature (22°C) to let the actin polymerize. The F-actin was spun down at 125,000*g* for 3 hours (Beckman Optima L-90K ultracentrifuge using SW-32Ti rotor at 32,000 rpm) forming a transparent pellet. The supernatant was removed (fig. S3, fraction 3), and the pellet was resuspended in 30 ml of G-actin buffer using a Dounce homogenizer (fig. S3, fraction 4). For depolymerization, the actin solution was dialyzed against 2 liters of G-actin buffer overnight (fig. S3, fraction 5). Actin was then polymerized at lower ionic strength by addition of ^1^/_10_ volume of 10× KMEH (low salt) [1× concentrations: 10 mM Hepes-KOH (pH 7.5), 20 mM KCl, 2 mM MgCl_2_, and 1 mM EGTA] for 2 hours at room temperature. This step reduces tropomyosin contamination (*68*). The filaments were again pelleted at 125,000*g* for 3 hours (Beckman Optima L-90K ultracentrifuge using SW-32Ti rotor at 32,000 rpm), the supernatant was discarded (fig. S3, fraction 6), and the pellet was resuspended in 10 ml of G-actin buffer using a Dounce homogenizer (fig. S3, fraction 7). For depolymerization, the actin solution was dialyzed for at least 1.5 days against 2 × 2 liters of G-actin buffer (fig. S3, fraction 8). The solution was finally spun at 4000*g* for 10 min (Beckman Allegra X-30R) to remove any residual insoluble material carried through the entire preparation. This G-actin stock solution was stored at 4°C until use but no longer than 3 weeks (fig. S3, fraction 9).

Before using actin for an experiment, it was further purified by SEC. For a small-scale preparation (used for the experiments in fig. S4, Fig. 3, and for bulk light scattering experiments), 3 × 175 μl of the actin stock were spun at 30 psi in a Beckman Airfuge using an A-100/18 rotor (~149,000*g*). A total of 450 μl were pooled from the supernatants and run on a Superdex 75 Increase 10/300 GL SEC column in G-actin buffer controlled by an Äkta Pure FPLC system (chromatogram; see fig. S4A). To prepare actin for the AMP-PNP experiments, the G-actin buffer for the SEC contained 0.2 mM AMP-PNP (Roche 10102547001) instead of ATP.

For larger-scale preparations (used for the majority of MP experiments), 4 ml of the actin stock was spun at 150,000*g* for 2 hours (Beckman Optima L-90K ultracentrifuge using SW-60Ti rotor at 38,200 rpm). The top 3 ml of the supernatant was run on a HiLoad 16/600 Superdex 75-pg SEC column in G-actin buffer controlled by an Äkta Pure FPLC system. Fractions from the right flank of the elution peak were pooled. After SEC, the actin stocks were stored on ice for a maximum of 1 week. Concentrations were determined by measuring the ultraviolet absorption at 290 nm (ε = 26,600 M^−1^ s^−1^) (*31*, *67*, *69*) corrected for scattering contributions by subtracting the absorption at 340 nm.

### Actin polymerization time courses recorded by bulk light scattering

Actin was diluted in G-actin buffer containing either 0.2 mM ATP or 0.2 mM AMP-PNP. The polymerization reaction was started by mixing with ^1^/_10_ volume of 10× KMEH. The scattering time course was recorded immediately with an approximate delay of 10 to 20 s after mixing. For the experiments shown in Fig. 2 (B and F) and figs. S10B and S14B, scattering of 20 μl of the reaction mix in a 1 cm × 1 cm quartz cuvette with a path length of 1.5 mm × 1.5 mm was detected using the Horiba FluoroMax-4 Spectrofluorometer, where the excitation and emission wavelengths were both set to 400 nm (2-nm slit width and 2-s read intervals with 0.1-s integration time) (*70*). The traces shown in Fig. 3A were recorded in the Varian Cary Eclipse Fluorescence Spectrometer using a 1 cm × 1 cm quartz cuvette with a path length of 2 mm × 1 cm and the same wavelengths (5.0-nm excitation slit width, 2.5-nm emission slit width, 2-s read intervals with 1.9-s integration time, and photomultiplier voltage set to high).

### Preparation of APTES coverslips

The negative surface charge of actin interferes with its adsorption to uncoated glass coverslips, i.e., the molecules hover across the surface rather than immediately binding to it. This behavior makes it difficult to reliably detect and quantify landing events in MP. We therefore modified the glass surface of our coverslips by introducing amino groups that are positively charged at the near neutral pH of our working buffers. The procedure is based on a protocol described in (*71*). Menzel microscope coverslips (50 mm × 24 mm, #1.5; Thermo Fisher Scientific) in a stainless steel holder were sonicated for 10 min in 2% (v/v) Hellmanex III in milliQ (18-megaohm ultrapure) water, 5 min in milliQ water, and 5 min in isopropanol. The coverslips and their holders were rinsed with milliQ water and blow-dried with a stream of nitrogen. The glass was activated by treatment with an oxygen plasma for 8 min (Diener Zepto One; 0.5 mbar of O_2_ pressure at 95% generator power). Directly afterwards, the coverslips were wetted in a beaker of acetone and then swirled in a solution of 2% (3-aminopropyl)-triethoxysilane (APTES; Sigma-Aldrich, A3648) in acetone for 1 min in a second beaker, allowing the APTES to adsorb to the negatively charged surface. Excess APTES was removed by transferring the coverslips through two beakers of fresh acetone. The solvent was removed from the coverslips by tapping the holder on a paper towel and letting the acetone evaporate. During this step, the surface of the coverslips usually turned cloudy as a sign of the APTES adsorption. The coverslips were then incubated for 1 hour at 110°C. As a sign of the covalent modification of the surface with APTES, the coverslips turned completely clear after the heat treatment. The APTES-coated coverslips were cleaned by sonication for 10 min with isopropanol and 5 min with milliQ water. Last, they were rinsed with milliQ water, blow-dried in a stream of nitrogen, and stored at room temperature protected from dust.

### MP experiments of actin solutions

The MP experiments were carried out on a custom-built mass photometer described in (*26*, *72*) with a 445-nm laser diode for illumination and 635 nm for focus stabilization. In initial test experiments, we noticed that the landing rate of actin on the APTES-modified glass surface is unusually high, most likely due to the strong attraction of the negatively charged molecules to the positively charged surface. Therefore, a measurement of actin landing at the concentrations needed in this study was not feasible, because individual landing events could not be sufficiently temporally isolated. We decided to include a dilution step immediately before measurement. Since a dilution would influence the observed equilibria, the solutions had to be measured as quickly as possible after dilution. The lowest time delay possible upon dilution was achieved by a setup in which 3-mm culture well gaskets (Grace Bio-Labs CW-50R-1.0) were attached to the coverslips, filled with a droplet of working buffer, and the focus position was adjusted. In this arrangement, the original actin solution could be added, and videos started immediately after the image had stabilized. This way, the usual time delay between sample addition and video start amounted to ~10 s. Depending on the original actin concentration, the dilutions ranged from 4-fold for 50 nM actin to 20-fold for 900 nM actin, making up a total volume of 40 μl in the gasket. As a control, we checked the dilution-induced changes in the distribution of 900 nM actin over the course of a 60-s video for consecutive 10-s video intervals (fig. S6). On the basis of these results, we considered the influence of the dilution step on the actin size distribution negligible.

A typical actin polymerization experiment was performed in the following way. The SEC-purified G-actin stock was diluted in G-actin buffer that had been kept at room temperature. A timer was started upon addition of ^1^/_10_ volume of 10× KMEH (high salt), adding up to a total volume of 50 μl and making up the actin working concentration (50 to 900 nM). After 1, 5, 10, or 15 min of incubation at room temperature, an aliquot was taken and added to a prepared droplet in a gasket as described in the previous paragraph, and a 1-min landing video was recorded at a frame rate of 1 kHz (then twofold averaged for saving, i.e., 500-Hz effective frame rate). As a control, the 10× KMEH (high salt) was replaced by G-actin buffer, demonstrating that the observed changes in the actin mass distribution are caused by the addition of polymerization inducing ions (K^+^ and Mg^2+^), as expected (fig. S7).

### Analysis of landing videos and mass calibration

The videos of proteins binding to the APTES-coated glass surface were analyzed with the software DiscoverMP (version 2.1.0, Refeyn Ltd). The small size of actin monomers of only 42 kDa approached the lower detection limit of our microscope. To determine the optimum frame averaging factor *navg* and filter thresholds (*T1* and *T2*) for quantitative detection of these species and to get an estimate of the percentage of correctly detected particles, we generated semisynthetic movies that used frames from a video recorded with G-actin buffer supplemented with ^1^/_10_ volume of 10× KMEH to simulate an experimental background and added simulated point spread functions as landing events that had the expected scattering contrast of actin monomers (contrast = 3.1 × 10^−3^). The model point spread function was the same function used to fit experimental landing events in DiscoverMP.

We then varied *navg* as well as *T1* and *T2*, ran the analysis procedure, and evaluated the number of true-positive and false-positive detections. To determine the maximum number of true-positive detections possible at the respective signal-to-noise ratio, we simulated 1000 frames with 100 landing events that were not allowed to overlap closer than 12 pixels spatially and 26 frames temporally. Based on this control simulation, we chose *navg* = 12, *T1* = 1.2, and *T2* = 0.15 to process the experimental videos. Using these parameters, the number of true-positive detection events of monomers in the simulation was (87.6 ± 1.7)% (mean ± SD of 5 simulations) and the number of false-positive detection events was (5.6 ± 1.5)%. For dimers (contrast = 5.6 × 10^−3^), a simulation with the same parameters gave (95.0 ± 2.4)% true-positive detection events and (0.6 ± 0.9)% false-positive detection events. The G-actin buffer video without simulated landing events did not produce any detection events.

Image contrast was converted into protein mass by determining the contrast of a set of mass standards with known mass in a working buffer droplet on a gasket and APTES surface (*26*). Here, protein mass standards included protein A (monomer: 42 kDa, Sigma-Aldrich, A4612), bovine serum albumin (monomer: 66 kDa, Fisher Scientific, BPE9700-100), and alcohol dehydrogenase (dimer: 73.5 kDa; tetramer: 147 kDa; Sigma-Aldrich, A8656). Figure S2 shows a typical mass calibration.

### Simulation of actin assembly

#### Kinetic scheme used for Sept/McCammon model

To compare our experimental data with the Sept/McCammon model (*35*), we generated a kinetic simulation tool based on the following polymerization scheme


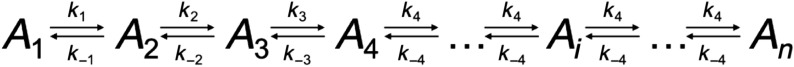


Here, *k*_1_, *k*_2_, *k*_3_, and *k*_4_ are the forward rate constants, and *k*_−1_, *k*_−2_, *k*_−3_, and *k*_−4_ are the backward rate constants. [*A*_1,2,3,4,*i*,*n*_] are the concentrations of monomers, dimers, …, etc. The dynamics of this system can be described by a set of ordinary differential equations (ODE) that determine the change in concentration for each species with time (*t*)

$$\begin{array}{cc} \frac{d[A]}{\mathrm{dt}}= & -2\;k_{1}{\left[A\right]}^{2}+2\;k_{-1}[A_{2}]-k_{2}[A_{2}][A]+k_{-2}[A_{3}]-\\ & k_{3}[A_{3}][A]+k_{-3}[A_{4}]-k_{4}[A_{4}][A]+k_{-4}[A_{5}]\ldots -\\ & k_{4}[A_{i}][A]+k_{-4}[A_{i+1}]\ldots -k_{4}[A_{n-1}][A]+k_{-4}[A_{n}] \end{array}$$ (1)

$$\frac{d[A_{2}]}{\mathrm{dt}}=k_{1}{\left[A\right]}^{2}-k_{-1}[A_{2}]-k_{2}[A_{2}][A]+k_{-2}[A_{3}]$$ (2)

$$\frac{d[A_{3}]}{\mathrm{dt}}=k_{2}[A_{2}][A]-k_{-2}[A_{3}]-k_{3}[A_{3}][A]+k_{-3}[A_{4}]$$ (3)

$$\frac{d[A_{4}]}{\mathrm{dt}}=k_{3}[A_{3}][A]-k_{-3}[A_{4}]-k_{4}[A_{4}][A]+k_{-4}[A_{5}]$$ (4)

$$\frac{d[A_{i}]}{\mathrm{dt}}=k_{4}[A_{i-1}][A]-k_{-4}[A_{i}]-k_{4}[A_{i}][A]+k_{-4}[A_{i+1}]$$ (5)

$$\frac{d[A_{n}]}{\mathrm{dt}}=k_{4}[A_{n-1}][A]-k_{-4}[A_{n}]$$ (6)

#### Kinetic scheme for ATP hydrolysis model and cation exchange model

Our new model used a scheme where actin oligomers larger than dimers may transition into a kinetically distinct state A′ with its own set of rate constants


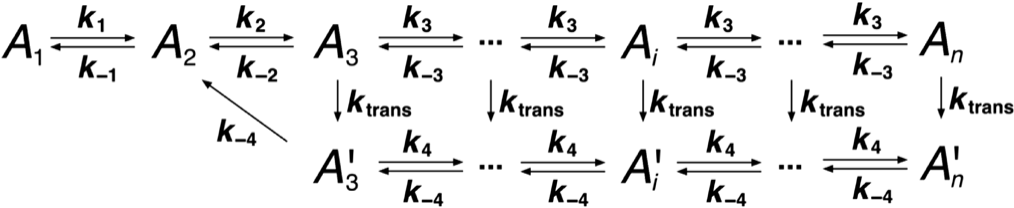


In this scheme, *k*_trans_ represent the rate constant of irreversible transition to state A′. The dynamics of this system were described by the following set of differential equations

$$\begin{array}{cc} \frac{d[A]}{\mathrm{dt}}= & -2\;k_{1}{\left[A\right]}^{2}+2\;k_{-1}[A_{2}]-k_{2}[A_{2}][A]+k_{-2}[A_{3}]-\\ & k_{3}[A_{3}][A]+k_{-3}[A_{4}]\ldots -k_{3}[A_{i}][A]+k_{-3}[A_{i+1}]\ldots -\\ & k_{3}[A_{n-1}][A]+k_{-3}[A_{n}]+k_{-4}[A_{3}^{\prime }]-k_{4}[A_{3}^{\prime }][A]+\\ & k_{-4}[A_{4}^{\prime }]\ldots -k_{4}[A_{i}^{\prime }][A]+k_{-4}[A_{i+1}^{\prime }]\ldots -k_{4}[A_{n-1}^{\prime }][A]+k_{-4}[A_{n}^{\prime }] \end{array}$$ (7)

$$\frac{d[A_{2}]}{\mathrm{dt}}=k_{1}{\left[A\right]}^{2}-k_{-1}[A_{2}]-k_{2}[A_{2}][A]+k_{-2}[A_{3}]+k_{-4}[A_{3}^{\prime }]$$ (8)

$$\frac{d[A_{3}]}{\mathrm{dt}}=k_{2}[A_{2}][A]-k_{-2}[A_{3}]-k_{3}[A_{3}][A]+k_{-3}[A_{4}]-k_{\text{trans}}[A_{3}]$$ (9)

$$\frac{d[A_{i}]}{\mathrm{dt}}=k_{3}[A_{i-1}][A]-k_{-3}[A_{i}]-k_{3}[A_{i}][A]+k_{-3}[A_{i+1}]-k_{\text{trans}}[A_{i}]$$ (10)

$$\frac{d[A_{n}]}{\mathrm{dt}}=k_{3}[A_{n-1}][A]-k_{-3}[A_{n}]-k_{\text{trans}}[A_{n}]$$ (11)

$$\frac{d[A_{3}^{\prime }]}{\mathrm{dt}}=-k_{-4}[A_{3}^{\prime }]-k_{4}[A_{3}^{\prime }][A]+k_{-4}[A_{4}^{\prime }]+k_{\text{trans}}[A_{3}]$$ (12)

$$\frac{d[A_{4}^{\prime }]}{\mathrm{dt}}=k_{4}[A_{3}^{\prime }][A]-k_{-4}[A_{4}^{\prime }]-k_{4}[A_{4}^{\prime }][A]+k_{-4}[A_{5}^{\prime }]+k_{\text{trans}}[A_{4}]$$ (13)

$$\frac{d[A_{i}^{\prime }]}{\mathrm{dt}}=k_{4}[A_{i-1}^{\prime }][A]-k_{-4}[A_{i}^{\prime }]-k_{4}[A_{i}^{\prime }][A]+k_{-4}[A_{i+1}^{\prime }]+k_{\text{trans}}[A_{i}]$$ (14)

$$\frac{d[A_{n}^{\prime }]}{\mathrm{dt}}=k_{4}[A_{n-1}^{\prime }][A]-k_{-4}[A_{n}^{\prime }]+k_{\text{trans}}[A_{n}]$$ (15)

For the ATP hydrolysis–based interpretation of this model (Fig. 2E), *k*_1_, *k*_2_, and *k*_3_ were set to the sum of barbed end and pointed end elongation rate constants (11.6 μM^−1^ s^−1^ + 1.3 μM^−1^ s^−1^ = 12.9 μM^−1^ s^−1^), and *k*_−2_ and *k*_−3_ were set to the sum of the corresponding barbed end and pointed end shortening rate constants (1.4 s^−1^ + 0.8 s^−1^ = 2.2 s^−1^), all reported by Pollard (*40*). In this interpretation, *k*_trans_ is equivalent to the ATP hydrolysis rate constant of actin subunits built into filaments (*44*). The dimer dissociation rate constant *k*_−1_, not previously reported, was adjusted manually. A list of all rate constants used for the ATP hydrolysis model is provided in table S2A.

For the cation exchange interpretation of this model (Fig. 3C), *k*_1_, *k*_2_, and *k*_3_ were set to the sum of the elongation rate constants at barbed and pointed end of actin filaments in buffer containing 0.2 mM CaCl_2_ and 100 mM KCl (9.5 μM^−1^ s^−1^ + 1.1 μM^−1^ s^−1^ = 10.6 μM^−1^ s^−1^), and *k*_−2_ and *k*_−3_ were set to the sum of the corresponding shortening rates (4.2 μM^−1^ s^−1^ + 0.8 μM^−1^ s^−1^ = 5.0 μM^−1^ s^−1^), all reported by Cooper *et al.* (*31*). The rate constants *k*_4_ and *k*_−4_ were again set to the abovementioned rates (12.9 μM^−1^ s^−1^ and 2.2 s^−1^, respectively), measured by Pollard (*40*) in 1 mM MgCl_2_ and 50 mM KCl. In this interpretation, *k*_trans_ is equivalent to the monomer activation rate reported by Cooper *et al.* (*31*). The dimer dissociation rate constant was adjusted manually. A list of all rate constants used for the cation exchange model is provided in table S2B.

Both systems of differential equations derived for kinetic schemes **1** and **2** can be solved by numerical integration, if they are restricted to a limited number of species *n*. We generated analysis pipelines in Python and Jupyter Lab that perform the integration using the scipy.integrate.odeint module (*73*, *74*).

As starting concentration of each species, we determined their respective counts from the experimental histogram at 1-min incubation time (chosen as *t*_0_). The counts of the first two to three species were determined by fitting a sum of Gaussians to the histogram in MATLAB (fig. S8A). The area under each Gaussian yielded the species count (fig. S8B). Species counts for the higher–molecular weight, less well-defined peaks were determined by counting detected molecules in 42-kDa spaced bins (fig. S8, A and B). The count of each species *N*_i_ simulated was multiplied by 1000 to increase statistics for the final output histogram (fig. S8D), and converted into the concentration [*A*_i_] (fig. S8F) using the known total concentration of actin [*A*]_total_

$$N_{\text{subunits},i}=N_{i}\times i$$ (16)

$${\left[A_{i}\right]}_{\text{subunits}}={\left[A\right]}_{\text{total}}\times \frac{N_{\text{subunits},i}}{\sum N_{\text{subunits},i}}$$ (17)

$$[A_{i}]=\frac{{\left[A_{i}\right]}_{\text{subunits}}}{i}$$ (18)

Here, *N*_subunits,i_ is the count of actin subunits accumulated in a species, *i* is the species index (e.g., three for a trimer), ∑*N*_subunits,*i*_ is the sum of subunit counts over all species, and [*A*_i_]_subunits_ is the concentration of a species scaled by the number of subunits in it.

With starting concentrations of each species determined in this way, a time course of the concentrations of each species was determined by numerical integration of the above differential equations (fig. S8G). For scheme 2, all species were assumed to be in the nonhydrolyzed state (top row in kinetic scheme) at *t*_0_. The output interval was chosen to be 30 s, and the maximum number of species *n* was set to 400.

To compare the solution of the numerical integration with our experimental data, the concentration output at *t*_0_ + 4 min, *t*_0_ + 9 min, and *t*_0_ + 14 min was first converted back into counts (fig. S8H) using the total subunit count ∑*N*_subunits,*i*_ at *t*_0_ (for simplicity here called *N*_total_)

$${\left[A_{i}\right]}_{\text{subunits}}=[A_{i}]\times i$$ (19)

$$N_{\text{subunits},i}=N_{\text{total}}\times \frac{{\left[A_{i}\right]}_{\text{subunits}}}{\sum {\left[A_{i}\right]}_{\text{subunits}}}$$ (20)

$$N_{i}=\text{round}(\frac{N_{\text{subunits},i}}{i})$$ (21)

For kinetic scheme 2, the counts of each species *A*_i_ and its respective *A*′_i_ were summed, since these states were indistinguishable by MP. Then, the species counts at each time point were converted into a molecule list where each species *A*_i_ has *N*_i_ occurrences in the list. Each molecule was designated a mass, based on the Gaussian fit of the *t*_0_ histogram (fig. S8J). The mass would be the Gaussian mean value of the corresponding species with added Poissonian noise that had an SD of the Gaussian fit. Species larger than those with a corresponding Gaussian fit were designated a multiple of 42 kDa with added Poissonian noise that had the standard deviation of the largest species’ Gaussian fit. The mass lists generated in this manner could be plotted as mass histograms and compared directly to the experimental mass histograms (fig. S8K).

We recently found that the relative landing rates of molecular species in MP scale with their diffusion speed (*26*). The lower diffusion speed of larger species causes them to land less frequently on the surface as compared to smaller ones. To correct for this effect, we determined the relative diffusion speed of each oligomer compared to monomers. For monomers to tetramers, we assumed spherical shape and scaled the diffusion coefficient of actin monomers [*D*_monomer_ = 7.9 × 10^−7^ cm^2^ s^−1^ in (*75*)] by the increase in mass according to

Doligomer,i=Dmonomer(Mmonomeri×Mmonomer)13,for i from 1 to 4 (22)

where *M*_monomer_ is the molecular mass of actin (42 kDa) and *i* is the number of subunits in the oligomer.

Since actin grows into filaments, however, it is not appropriate to model the higher-order oligomers as spherical particles. For oligomers larger than tetramers, we therefore calculated the diffusion speeds of rods that have a diameter *d* of 7 nm and length *L* of *i* × 2.7 nm (*46*, *47*) based on the model by Tirado *et al.* (*76*)

$$D_{\text{rod},i}=\frac{k_{B}T}{3\pi \eta L}\;(\text{ln}p+v),\text{for}\;i\;\text{from}\;5\;\text{to}\;n$$ (23)

p=Ld (24)

$$v=0.312+\frac{0.565}{p}-\frac{0.1}{p^{2}}$$ (25)

where *k*_B_*T* is the Boltzmann factor at 20°C and η is the viscosity of water (10^−3^ Pa s).

The starting count for the simulation of each species at *t*_0_ = 1 min was then scaled up (fig. S8E) before the simulation to adjust to the correct relative amounts in the sample (*N*_i,corrected_ = *N*_i_ × *D*_monomer_/*D*_oligomer/rod,*i*_) and after the simulation at the respective time points it was scaled back down (fig. S8, I to J) to compare to the experimental histograms. Figure S9A shows the magnitude of diffusion coefficients *D*_oligomer,*i*_ and *D*_rod,*i*_ calculated for different oligomer sizes *i* according to Eqs. 22 and 23, respectively. The effect on the resulting simulated mass histograms is illustrated in fig. S9B. In addition, we performed this type of upscale-downscale correction for the monomer count *N*_1_ due to the imperfect detection efficiency described in the “Analysis of landing videos and mass calibration” section (i.e., *N*_1,corrected_ = *N*_1_/0.876; fig. S8, E and I to J).

### Simulation of bulk scattering curves

We wanted to assess whether our kinetic models resemble the polymerization behavior of an actin solution in a bulk light scattering experiment. For this, we performed an MP experiment with the G-actin stock used for the bulk light scattering experiment and determined the individual species concentrations from its mass distribution as described in the previous section. Using these as starting conditions, we determined the concentration time course of each species by numerical integration of the respective model’s differential equations (see above) at a total actin concentration of 2 μM as in our bulk scattering experiment. Here, we used a maximum number of species of 2000 and an output interval of 2 s, equal to the measurement interval of the experiment.

The scattering signal of an actin solution is composed of the summed scattering contributions of all individual species. The scattering contribution of an actin oligomer scales with its concentration and squared molecular mass, i.e., number of subunits (*77*). Depending on the spectrometer’s sensitivity, the scattering contribution of small species will fall below the lower detection limit and only the signal of large species may contribute to the signal. On the basis of these considerations, we calculated the scattering signal from the individual species concentrations at each time point of the simulation according to

$$\text{Scattering signal}=s\times \sum_{i=\text{sds}}^{n=2000}i^{2}[A_{i}]$$ (26)

[*A*_i_] is the concentration of the actin oligomer with *i* subunits, sds is the smallest detectable species, *n* is the largest simulated species, and *s* is a scaling factor. Since the smallest detectable species was unknown for the photodetector, it was handled as an optimization parameter (fig. S10). Also, the exact scaling *s* between the simulated scattering signal and the detector count in our spectrometer was not known. Therefore, we scaled our simulated scattering time course according to the average start and end scattering levels of the experimental curves. This simulation was implemented as a Python routine.

### MCMC parameter analysis and model fitting

The above-described custom software tools were subsequently used to jointly fit the experimental MP histograms and separately acquired bulk light scattering data (both acquired as a function of time) to the different kinetic models in a Bayesian framework. To reduce computational complexity, all parameters other than the rate equations were fixed, and the numba package was used together with the llvm compiler (*78*) to just-in-time compile the computationally expensive ODE integration procedures to run as optimized machine code, using AVX and similar CPU extensions together with compiler fastmath optimization that may lead to a negligible decrease in numerical precision.

As a brief refresher, we desire to use Bayesian approaches to derive the posterior probability densities and credible intervals for the set of parameters of interest (i.e., rate constants) θ of the models described in the “Simulation of actin assembly” section, given the observed data. We furthermore wish to compute information criteria such as the Akaike information criterion (AIC) and Bayesian information criterion (BIC) to provide an information theoretically sound framework for model selection. Accordingly, we are interested in the posterior probability density function (pdf) *p*(θ∣*D*) given by $p(\theta |D)=\frac{L\;p(\theta )}{\int p(D|\theta )p(\theta )\;\mathrm{d\theta}}$, where ℒ is the likelihood function, *p*(θ) is the prior distribution reflecting degrees of belief in θ, and ∫*p*(*D*∣θ)*p*(θ) dθ is a term commonly called the evidence and that is commonly interpreted to represent a normalizing constant. The likelihood ℒ represents the probability of obtaining the data *D* given a physical model of both the data and its uncertainties. For numerous reasons, not least of which that ℒ is typically very small in absolute terms, it is common to work with the log likelihood function and compute the log posterior. Here, we defined a joint likelihood function under the assumption of Gaussian errors for bulk light scattering data and the MP data, combining measurements from different experiments as is performed in the mathematical sciences. Here, as the scale of measurements is very different, it is necessary to include an (unknown) relative measurement scale term $\sigma_{j}^{2}$ to marginalize over: Errors within each technique are approximately independent and constant, and therefore, it is not required to explicitly determine them as they would just form a fractional alteration of $\sigma_{j}^{2}$. This gives a final expression for the likelihood of

$$-\text{ln}\;L=\sum_{j=\text{BLS},\text{MP}}2N_{j}\;\text{ln}\;\sigma_{J}+N_{j}\;\text{ln}\;2\pi +(\sum_{i=1}^{N_{j}}\frac{{\left(y_{i,j}-f_{i,j}(\theta )\right)}^{2}}{2\sigma_{j}^{2}})$$ (27)

where *N_j_* is the total number of points *y*_*i*,*j*_ in the *j*th dataset considered (containing *N*_BLS_ = 1800 data points and *N*_MP_ = 11,200 mass bins) and *f*(θ) is the simulation results at that point. Two brief things deserve noting. First, the fit here is to the midpoint of the bins of the histogram. We note that although it is often desirable to use an explicit form of the distribution for the model likelihood of a histogram if it is available, there is a long history of Poissonian maximum likelihood estimation in the context of either astronomy, particle physics, or fluorescence microscopy, where (if bin counts are large) it corrects for a small bias otherwise present in least-squares routines and is usually appropriate for the analysis of count data, if the probability of observing the entities studied is constant per unit time (*79*, *80*). However, in this case, that is clearly not true: The theoretical distribution does not go to zero the limit as the polymer mass becomes arbitrarily large in the limit as time goes to infinity; we therefore have adopted the so-called Nyman χ^2^ given. Second, note that the two effective marginal parameters $\sigma_{j}^{2}$ can be rescaled (with knowledge of the fixed *N_j_*) to effectively represent the relative weighting of both sources of information and the joint probability of observing them, given knowledge of θ.

For each model, we used a well-tested Python implementation of the affine-invariant ensemble sampler for MCMC methods (*81*) to create an ensemble of 100 parameter-space samplers distributed in a Gaussian ball around the previously published empirically determined rate constants. Priors were set to be uninformative, that is, uniform on the interval [0, *I_m_*] where *I_m_* represents a large number (10^5^) times the previously published rate constant for that particular rate constant. This effective bounding of the parameter space to a subset of the upper-half plane was chosen primarily out of considerations of computational complexity but additionally because it was considered a priori that the prior likelihood of previously published results being incorrect by several orders of magnitude was low enough to be considered zero.

Owing to the computational complexity of running each full simulation, and hence computing the likelihood function, it was necessary to run the ensemble sampler massively in parallel using MPI and the Arcus-b supercomputing cluster. For each sampler, candidate move proposals were chosen at random using either the differential evolution scheme (*82*) (80% probability) modified form of the “snooker” scheme (*83*) (20% probability), which aims to shoot proposal walkers through local minima in probability space and is reported to be 5 to 26 times more efficient than differential evolution alone, at the cost of more message-passing overhead between independent chains.

Approximately 250,000 individual samples were generated for each dataset. After computation, the sampler was checked for convergence by analysis of its autocorrelation time as proposed previously (*84*), and AICs and BICs were computed. Corner plots [i.e., plots of the one- and two-dimensional projections of the multidimensional posterior pdf *p*(θ)] for the parameters, which easily illustrate the posterior pdfs, were additionally computed. It was found that, as expected, the marginalized nuisance parameters $\sigma_{j}^{2}$ rapidly took values that corresponded to the approximate variance of the noise of the experimental data (~1740 arbitrary units for the bulk light scattering data; ~0.003 for all MP data). To reduce the dimensionality of the fit, we therefore fixed these parameters at these values and explored the parameter space of the rate constants independently; we note that this is analogous to a convex optimization of both terms simultaneously.

The results of our MCMC sampling for the ATP hydrolysis and Sept/McCammon model are illustrated in fig. S13 (A and B), respectively. Since each model has an *N*-dimensional parameter space with *N* being the number of rate constants, all of which are varied per iteration, we represented this by plotting the parameter space as a collection of two-dimensional planes along two rate constant axes. Each point in these two-dimensional planes represents an iteration of the MCMC walk through parameter space. Therefore, the point density, here represented in a series of two-dimensional histograms, is a measure of how well the model describes the data with respect to a pair of parameter values. Accordingly, the global maxima throughout all plots represent the best-fit parameters found for the model and are equivalent to the maximum likelihood value for the fit.
