# Supplementary material for: Direct observation of the molecular mechanism underlying protein polymerization
Source: Sci Adv. 2022 Aug 31;8(35):eabm7935. doi: 10.1126/sciadv.abm7935 (PMC9432825; doi:10.1126/sciadv.abm7935)
Supplement: Supplementary file 1 — Figs. S1 to S16 Tables S1 to S3 [file sciadv.abm7935_sm.pdf]

Science Advances
AAAS

Supplementary Materials for

Direct observation of the molecular mechanism underlying protein polymerization

Nikolas Hundt *et al.*

Corresponding author: Philipp Kukura, philipp.kukura@chem.ox.ac.uk

Sci. Adv. **8**, eabm7935 (2022)
DOI: 10.1126/sciadv.abm7935

This PDF file includes:

Figs. S1 to S16
Tables S1 to S3

Supplementary Figures

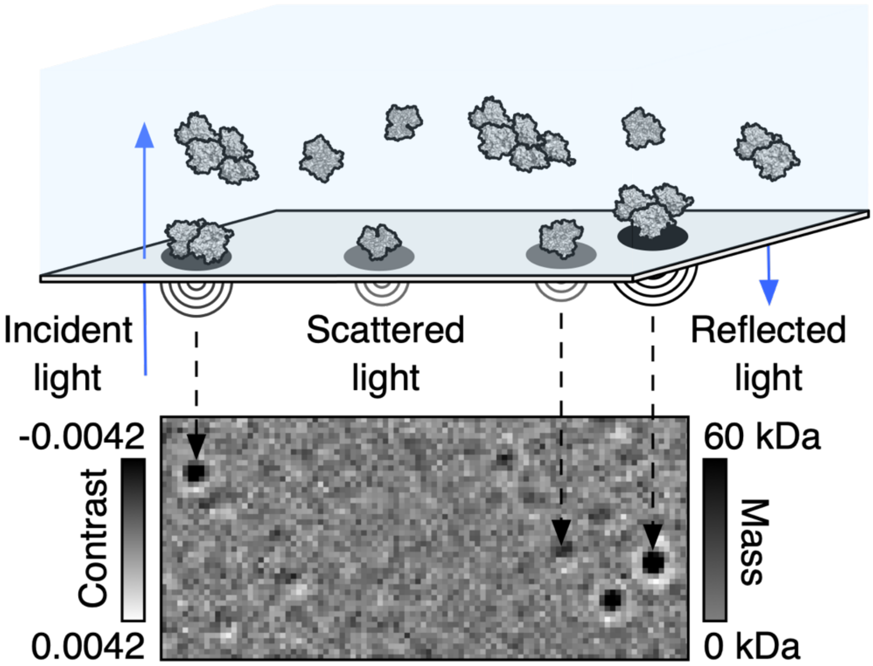

Figure S1 – Principle of detecting and quantifying actin oligomers by mass photometry

A glass coverslip holds a drop of actin solution and is illuminated from the bottom. Reflected light from the glass-water interface and scattered light from landing actin molecules are collected through an objective and directed onto a detector. The interferometric scattering signal intensity of actin oligomers is proportional to their molecular mass. The signal magnitude of each landing molecule is quantified and plotted in a mass histogram revealing the size distribution of molecule species in solution.

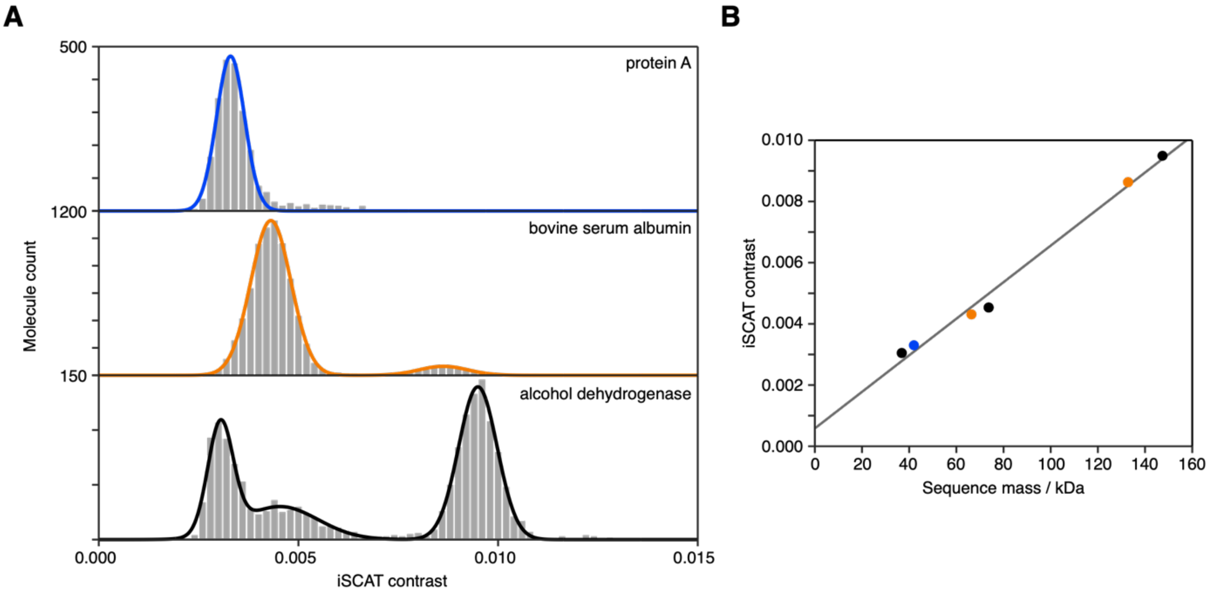

Figure S2 – iSCAT contrast to mass relation

(A) iSCAT contrast histograms determined for standard proteins with known sequence mass in F-actin buffer and corresponding Gaussian fits: protein A (blue) 1mer – 42.0 kDa (n = 2,326 particles); BSA (orange) 1mer/2mer – 66.4/132.8 kDa (n = 7,800 particles); ADH (black) 1mer/2mer/4mer – 36.9/73.7/147.4 kDa (n = 1,638 particles). **(B)** Species mean contrast determined from Gaussian fits in A plotted versus protein sequence mass. Actin contrast was converted to mass based on the grey calibration line.

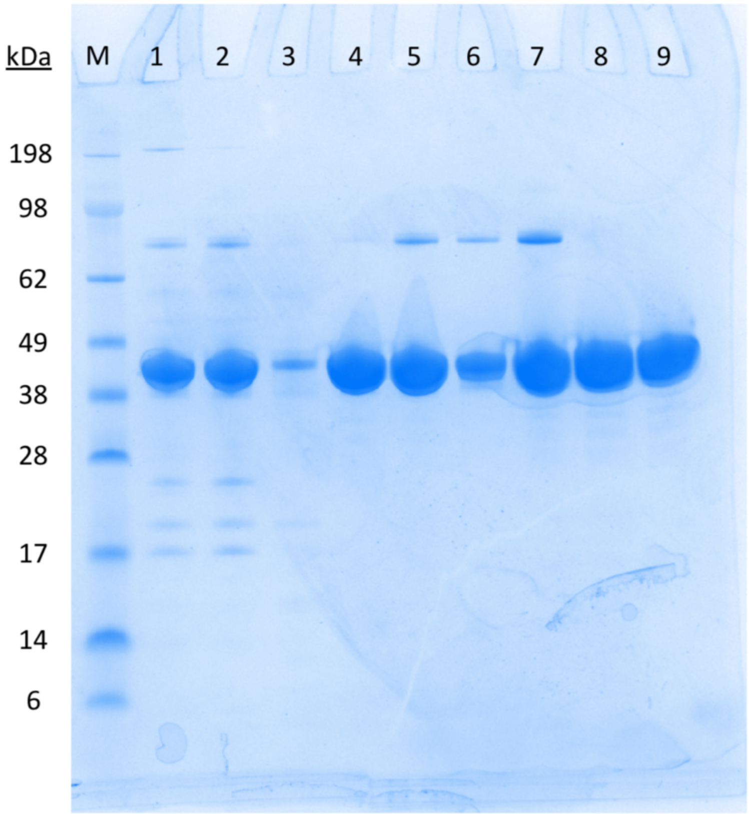

Figure S3 – SDS-PAGE of samples taken during actin purification

M – Protein marker; 1 – acetone powder suspension; 2 – cleared acetone powder suspension; 3 – supernatant of spin after first round of polymerization; 4 – resuspended pellet after first round of polymerization; 5 – after dialysis in G-actin buffer; 6 – supernatant of spin after second round of polymerization; 7 – resuspended pellet after second round of polymerization; 8 – after dialysis in G-actin buffer; 9 – purified actin used for size exclusion chromatography.

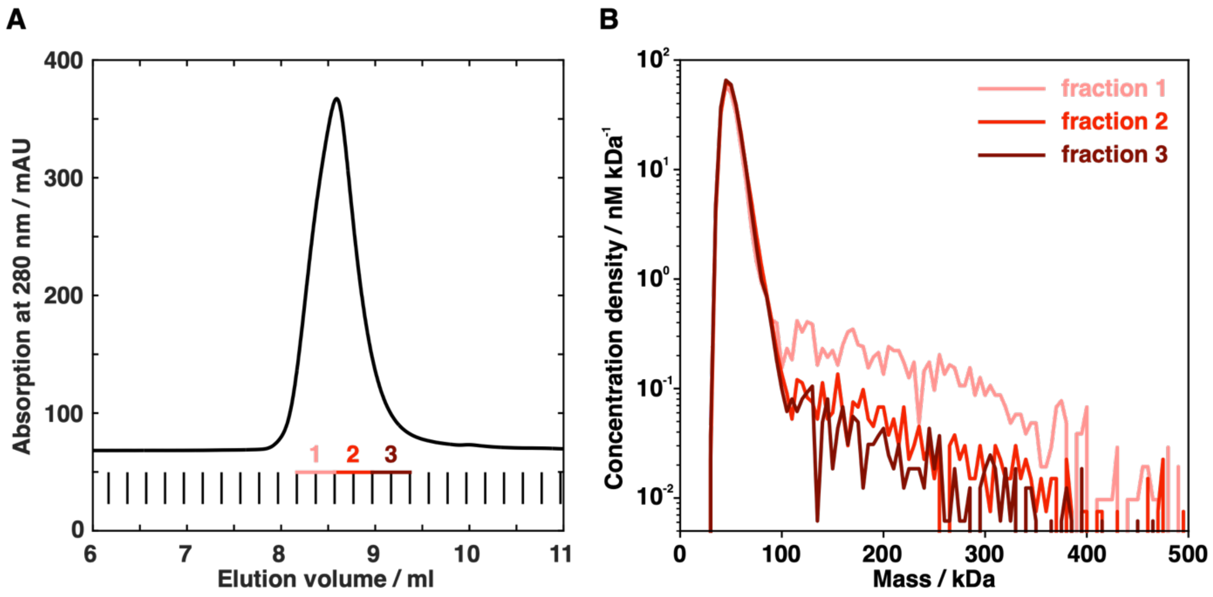

Figure S4 – Comparison of G-actin size exclusion chromatography with mass photometry

(A) Typical size exclusion chromatogram of G-actin using a Superdex 75 Increase 10/300 GL column. Grey vertical bars indicate starts of fractions. The indicated fractions were pooled and analyzed using mass photometry. **(B)** Corresponding MP mass distributions of the SEC fractions in A, each at 300 nM total actin. Particle numbers pooled from 2 technical replicates: $n_{fraction\ 1}$ = 22,782, $n_{fraction\ 2}$ = 31,946, $n_{fraction\ 3}$ = 39,932.

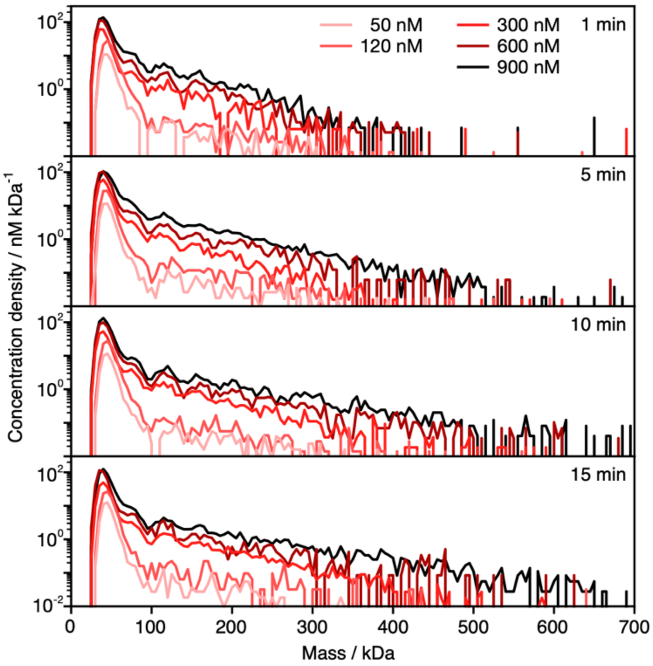

Figure S5 – Experimental time course of actin mass distributions under polymerizing conditions for five actin concentrations

Complete set of actin concentrations measured in the experiment described in Fig. 1C, which only shows 120 and 900 nM for clarity. Particle numbers pooled from 3-5 technical replicates: 50 nM - $n_{1\ min}$ = 4,372, $n_{5\ min}$ = 4,619, $n_{10\ min}$ = 4,991, $n_{15\ min}$ = 4524; 120 nM - $n_{1\ min}$ = 7,348, $n_{5\ min}$ = 5,797, $n_{10\ min}$ = 7,017, $n_{15\ min}$ = 2,973; 300 nM - $n_{1\ min}$ = 3,708, $n_{5\ min}$ = 14,831, $n_{10\ min}$ = 11,841, $n_{15\ min}$ = 23,009; 600 nM - $n_{1\ min}$ = 9,037, $n_{5\ min}$ = 7,135, $n_{10\ min}$ = 12,305, $n_{15\ min}$ = 5,213; 900 nM - $n_{1\ min}$ = 9,000, $n_{5\ min}$ = 29,314, $n_{10\ min}$ = 14,211, $n_{15\ min}$ = 19,668.

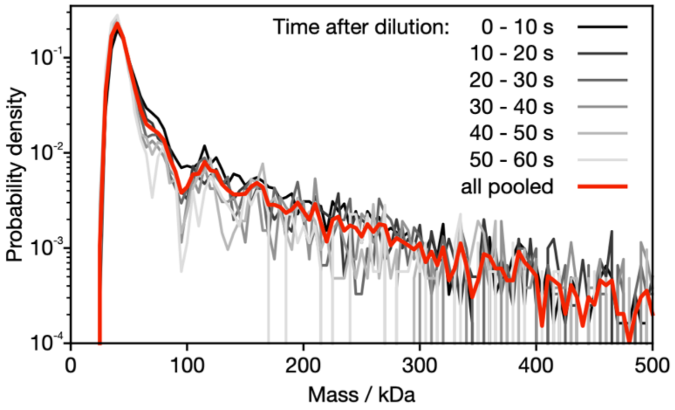

Figure S6 – Influence of gasket dilution step on actin mass distribution

Size distributions of 900 nM actin polymerized for 15 min resulting from events recorded in consecutive 10 s video intervals (grey shades) after 20-fold dilution in a droplet placed on a gasket as compared with the size distribution resulting from all events pooled. Particle numbers pooled from 5 technical replicates: 0 - 10 s: n = 6,160; 10 - 20 s: n = 4,008; 20 - 30 s: n = 3,037; 30 - 40 s: n = 2,617; 40 - 50 s: n = 2,080; 50 - 60 s: n = 1,766; all pooled: n = 19,668.

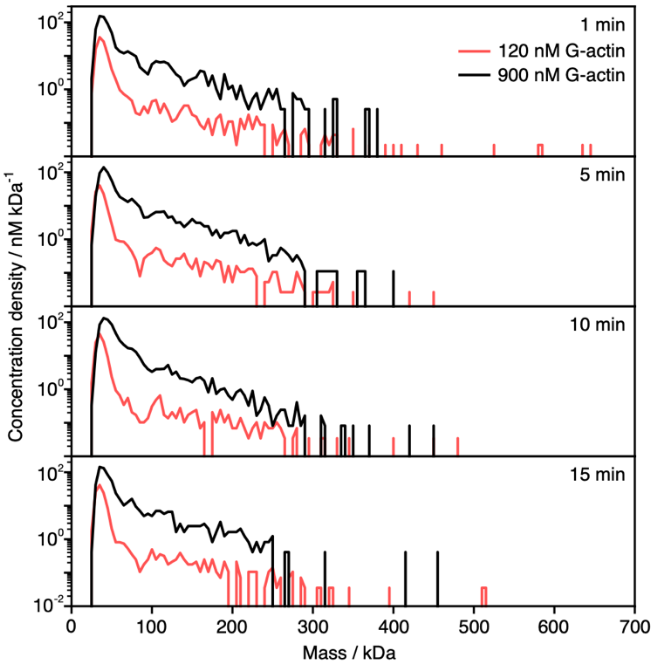

Figure S7 – Experimental time course of G-actin mass distributions

Evolution of the oligomeric distribution at 120 nM and 900 nM total actin as a function of time in a similar experiment as in Fig. 1C without adding KCl and $MgCl_2$ (added same volume of G-actin buffer instead). Particle numbers pooled from 1-3 technical replicates: 120 nM - n_{1min} = 4,810, $n_{5\ min}$ = 4,270, $n_{10\ min}$ = 3,412, $n_{15\ min}$ = 3,211; 900 nM - n_{1min} = 3,211, $n_{5\ min}$ = 5,541, $n_{10\ min}$ = 8,039, $n_{15\ min}$ = 1,567.

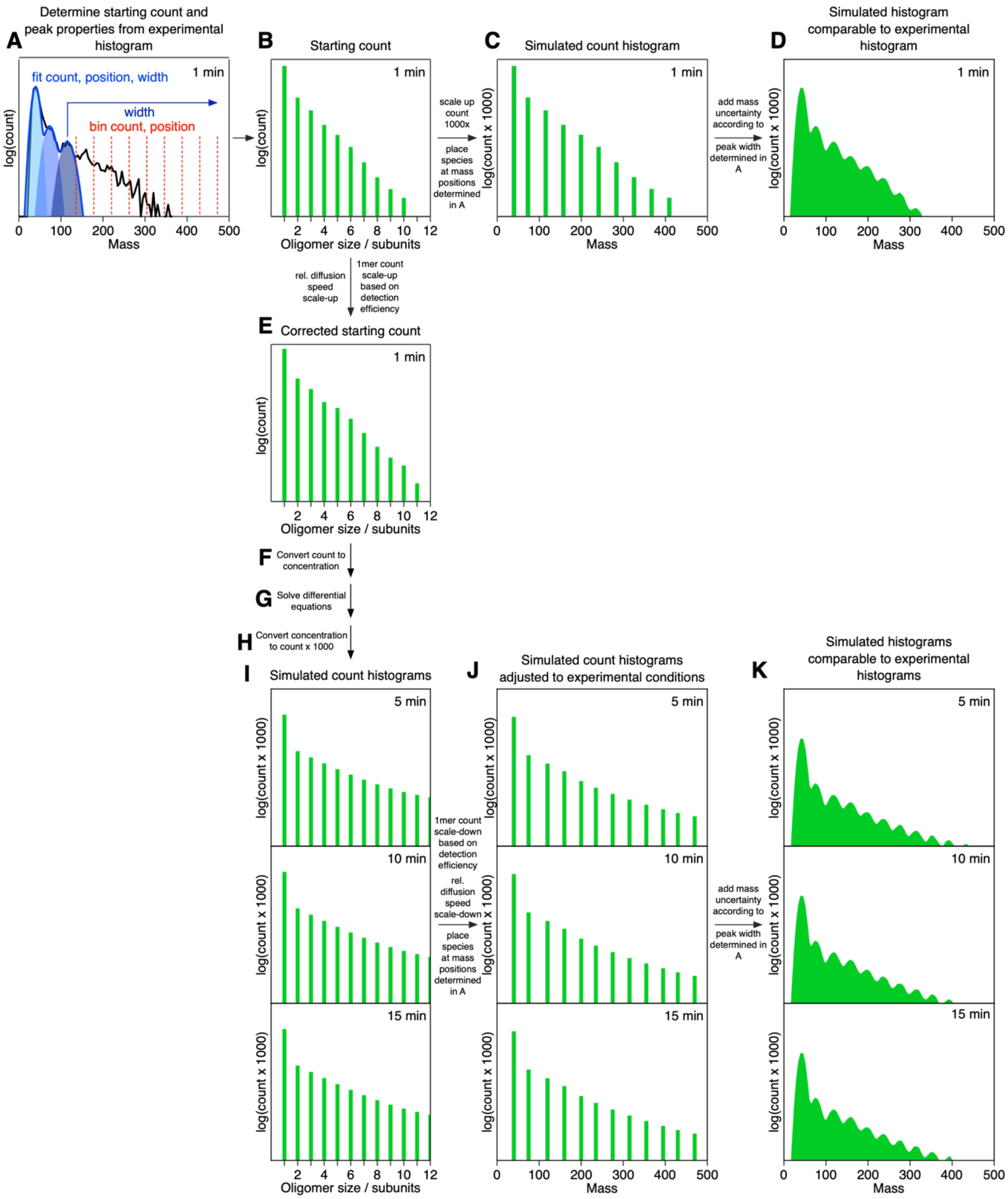

Figure S8 – Schematic summary of the workflow for generating simulated mass distributions

This figure represents a graphical illustration of the procedure described in detail in the methods section *Simulation of actin assembly*.

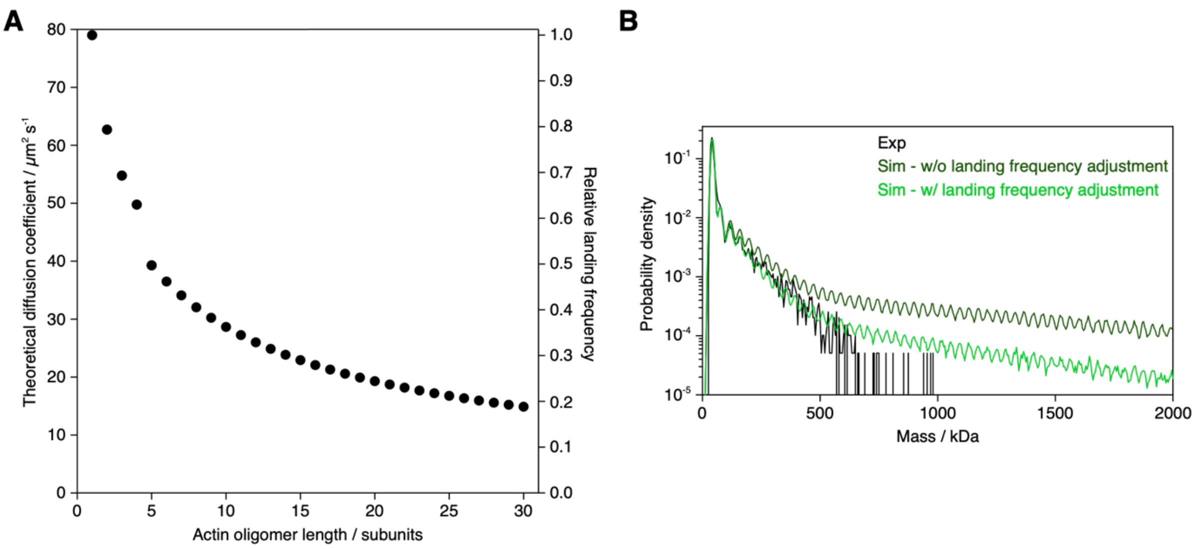

Figure S9 – Effect of oligomer diffusion speed on the actin mass distribution

(A) Left axis: Diffusion coefficient of actin oligomers as a function of their length calculated based on the procedure described in the section *Simulation of actin assembly*. Right axis: Corresponding drop of landing frequency expected in a mass photometry experiment. **(B)** Effect of oligomer length-dependent drop of landing frequency shown in A on simulated mass distributions. Exemplary distributions are shown for a simulation based on the ATP hydrolysis model with literature rate constants (Table S2A) at 900 nM actin and 15 min after polymerization start. Black: experimental distribution, dark green: simulated distribution without landing frequency adjustment, green: with landing frequency adjustment.

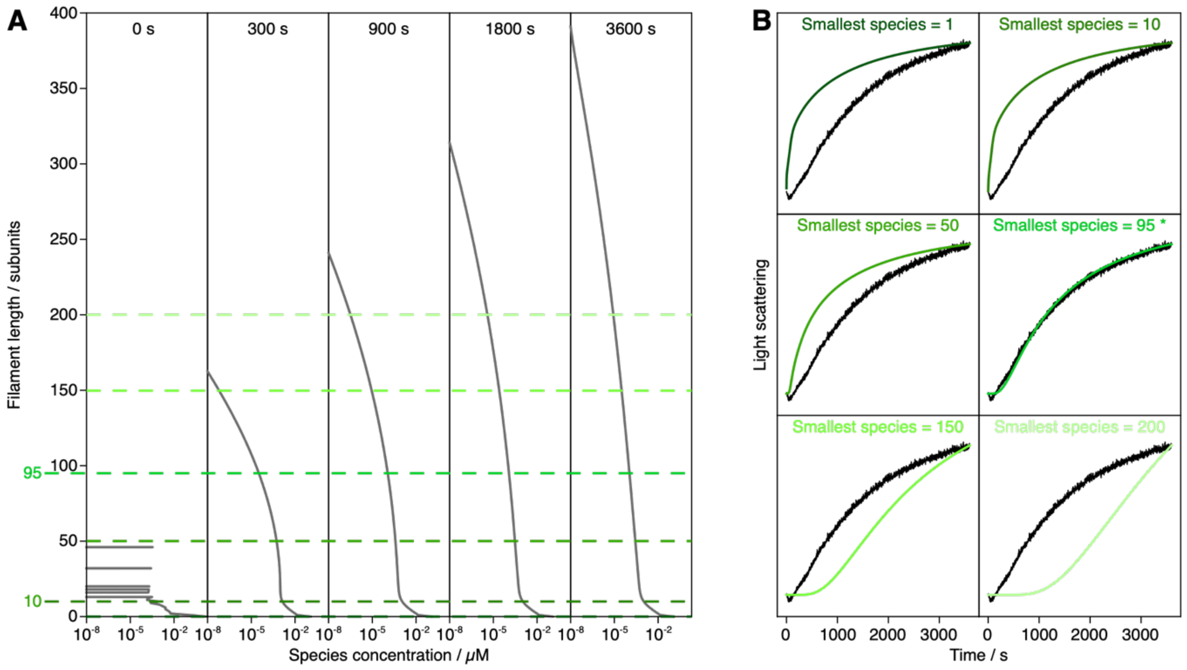

Figure S10 – Influence of smallest detectable species parameter on the outcome of simulated bulk scattering time courses

(A) Simulated time course of filament length distribution based on the ATP hydrolysis model with literature rate constants for a solution of 2 µM actin. Green dashed lines indicate the locations of the cut-off values for detection presumed in the bulk light scattering simulations in B. **(B)** Theoretical bulk light scattering time courses (green) calculated as described in the section *Simulation of bulk scattering curves*, using the indicated smallest species as detection cut-off. Asterisk: smallest detectable species parameter chosen as optimum to describe the experimental scattering trace at 2 µM actin (black).

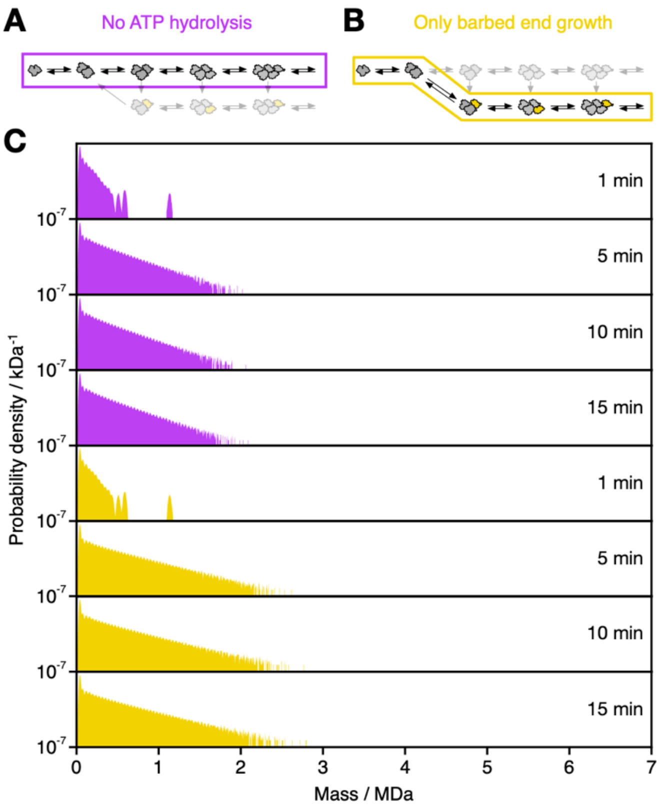

Figure S11 – The transition into kinetically more stable species is required for net filament growth.

(A) Kinetic scheme allowing only the top reaction pathway in Fig. 2E. **(B)** Kinetic scheme allowing only the bottom reaction pathway in Fig. 2E. **(C)** Time course of simulated mass distributions for 900 nM polymerizing actin based on the kinetic models in A (purple) and B (gold).

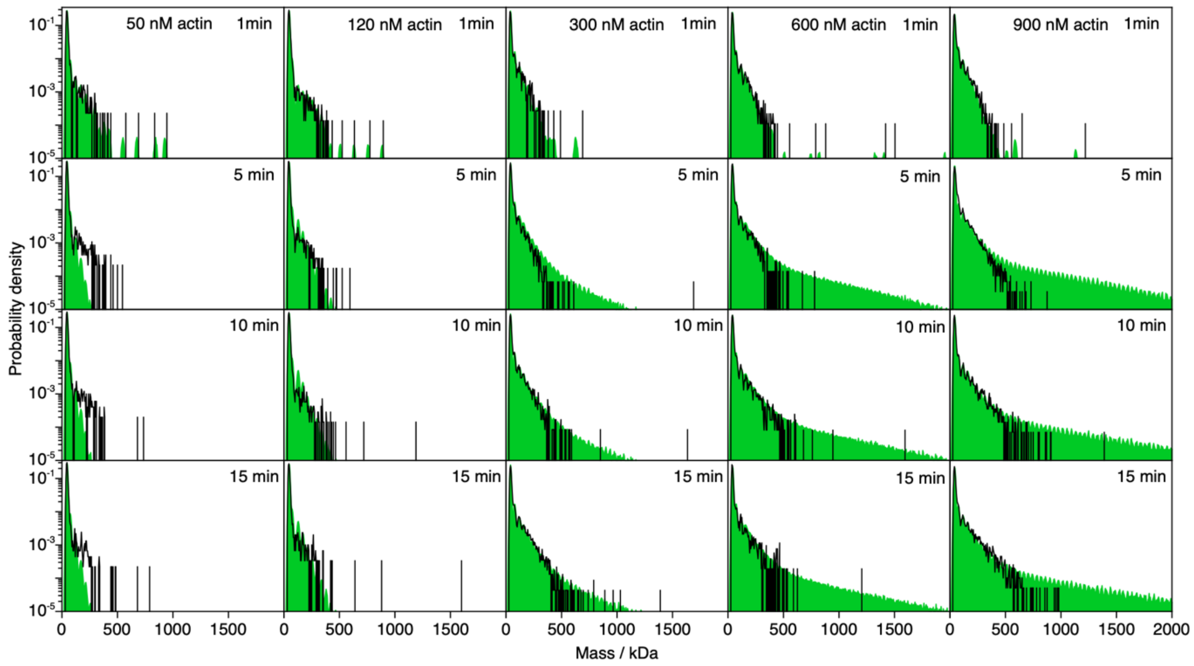

Figure S12 – Comparison of all experimental mass distributions with simulated distributions based on the ATP hydrolysis model with literature rate constants

Black lines represent mass distributions determined with mass photometry at the indicated time points and actin concentrations after inducing actin polymerization by addition of 100 mM KCl and 2 mM $MgCl_2$. Green distributions represent simulated mass histograms based on the ATP hydrolysis model depicted in Fig. 2E using the literature rate constants in Table S2A.

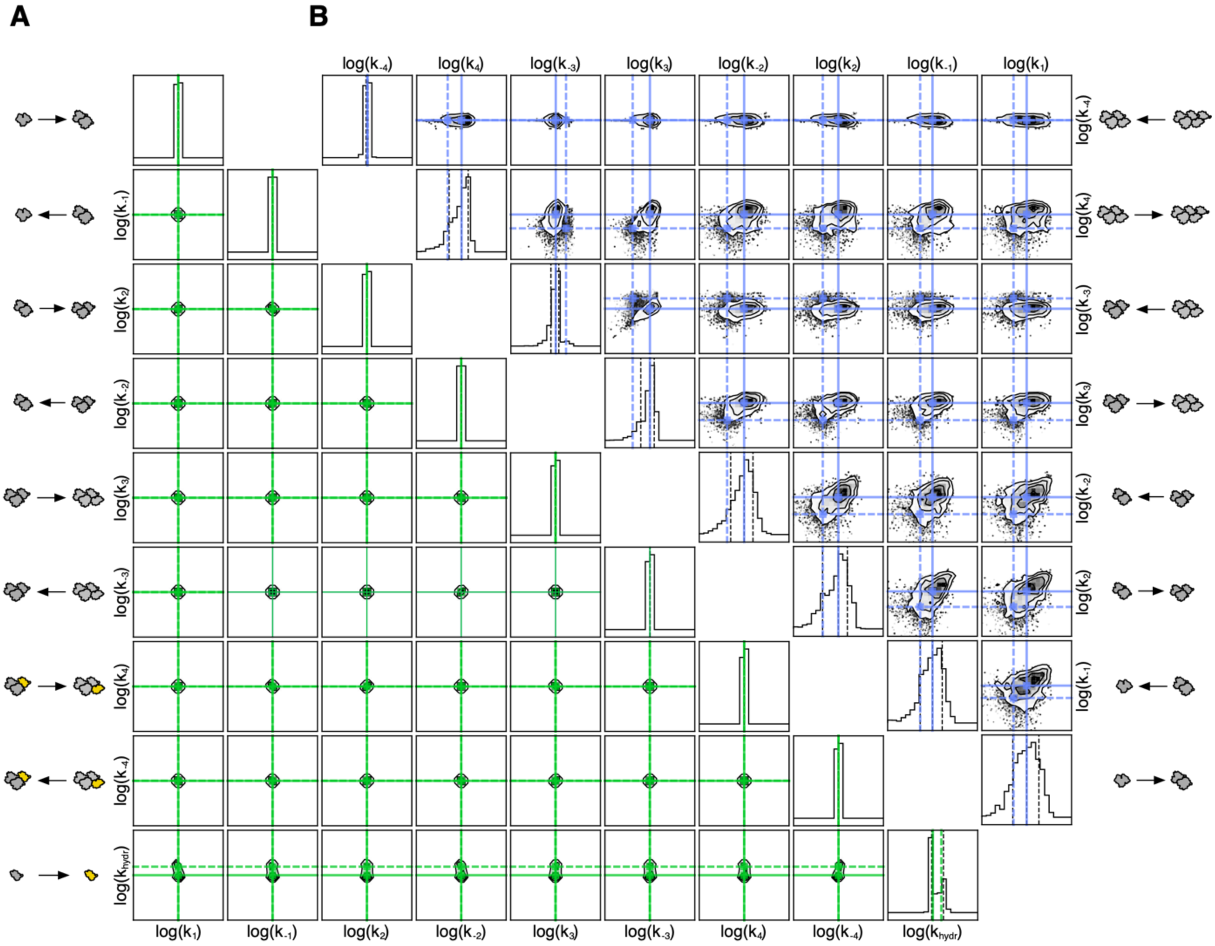

Figure S13 – Markov-Chain Monte Carlo sampling as validation of ATP-hydrolysis model for actin assembly

(A) Markov-Chain Monte Carlo sampling plots for the ATP-hydrolysis model. **(B)** Markov-Chain Monte Carlo sampling plots for the Sept/McCammon model. Shown are two-dimensional slices through parameter space along the indicated logarithmic rate constant axes (cartoons indicate the corresponding reaction steps). The histograms quantify the MCMC iteration density at the respective pair of rate constants. Rate constant axes are centered around the respective optimum rate constant (A: green crosshairs, B: blue crosshairs) given in Table S3 ± 4 orders of magnitude. Dashed crosshairs indicate the literature rate constants in Tables S1A and S2A.

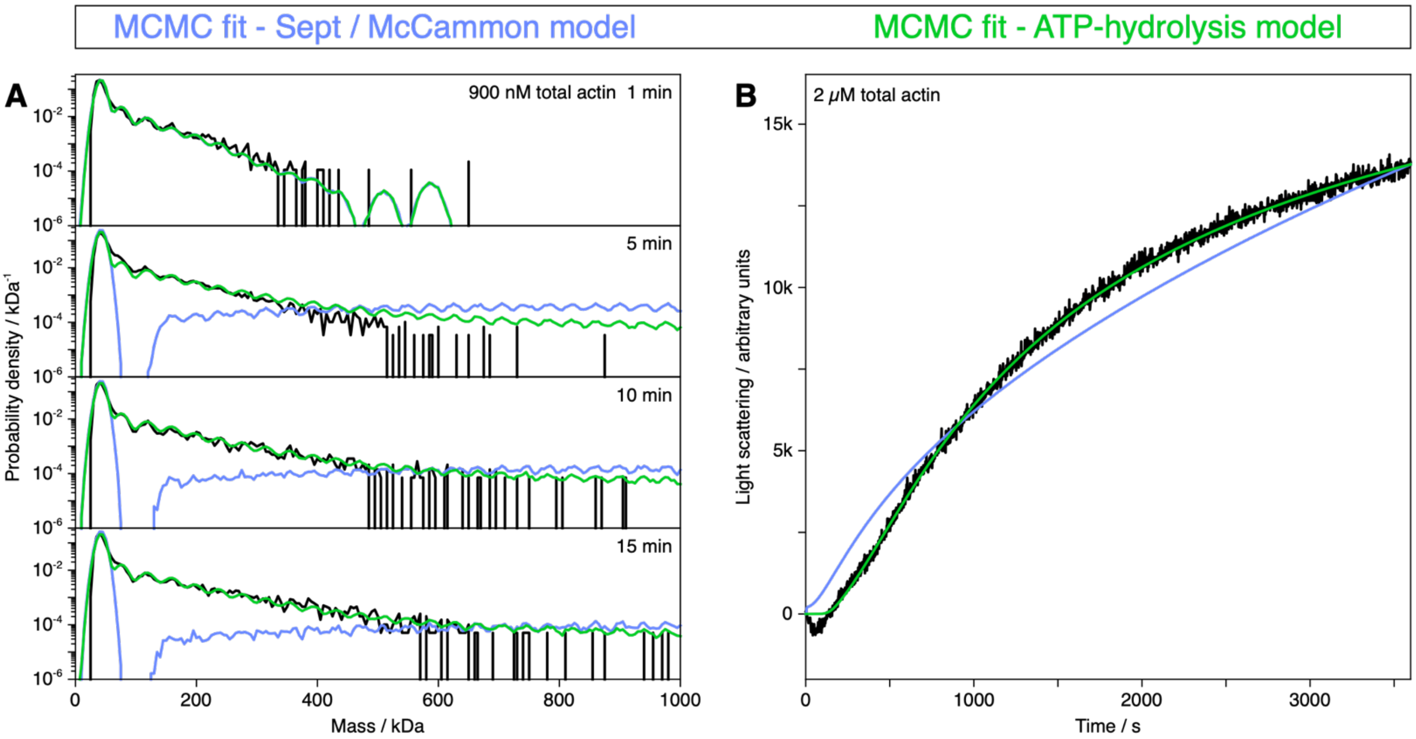

Figure S14 – Best fit results based on Markov-Chain Monte Carlo optimized rate constants for the two tested models

(A) Comparison of experimental (black) and simulated (colored) MP mass distributions at 900 nM total actin 1, 5, 10 and 15 min after initiation of polymerization. **(B)** Comparison of experimental (black) and simulated (colored) bulk light scattering time courses at 2 μM total actin. Simulations were based on the MCMC optimized rate constants summarized in Table S3. Blue – Sept/McCammon model shown in Fig. 2A; green – ATP-hydrolysis model shown in Fig. 2E.

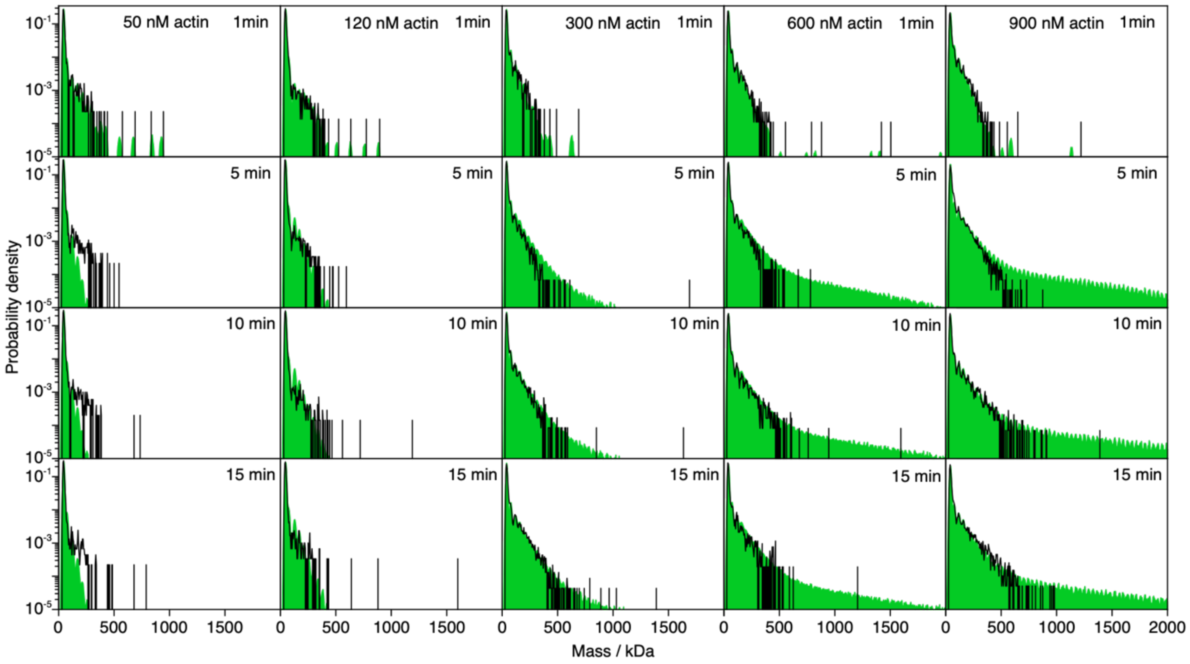

Figure S15 – Comparison of all experimental mass distributions with simulated distributions based on the ATP hydrolysis model with MCMC-optimized rate constants

Black lines represent mass distributions determined with mass photometry at the indicated time points and actin concentrations after inducing actin polymerization by addition of 100 mM KCl and 2 mM $MgCl_2$. Green distributions represent simulated mass histograms based on the ATP hydrolysis model depicted in Fig. 2E using MCMC-optimized rate constants in Table S3B.

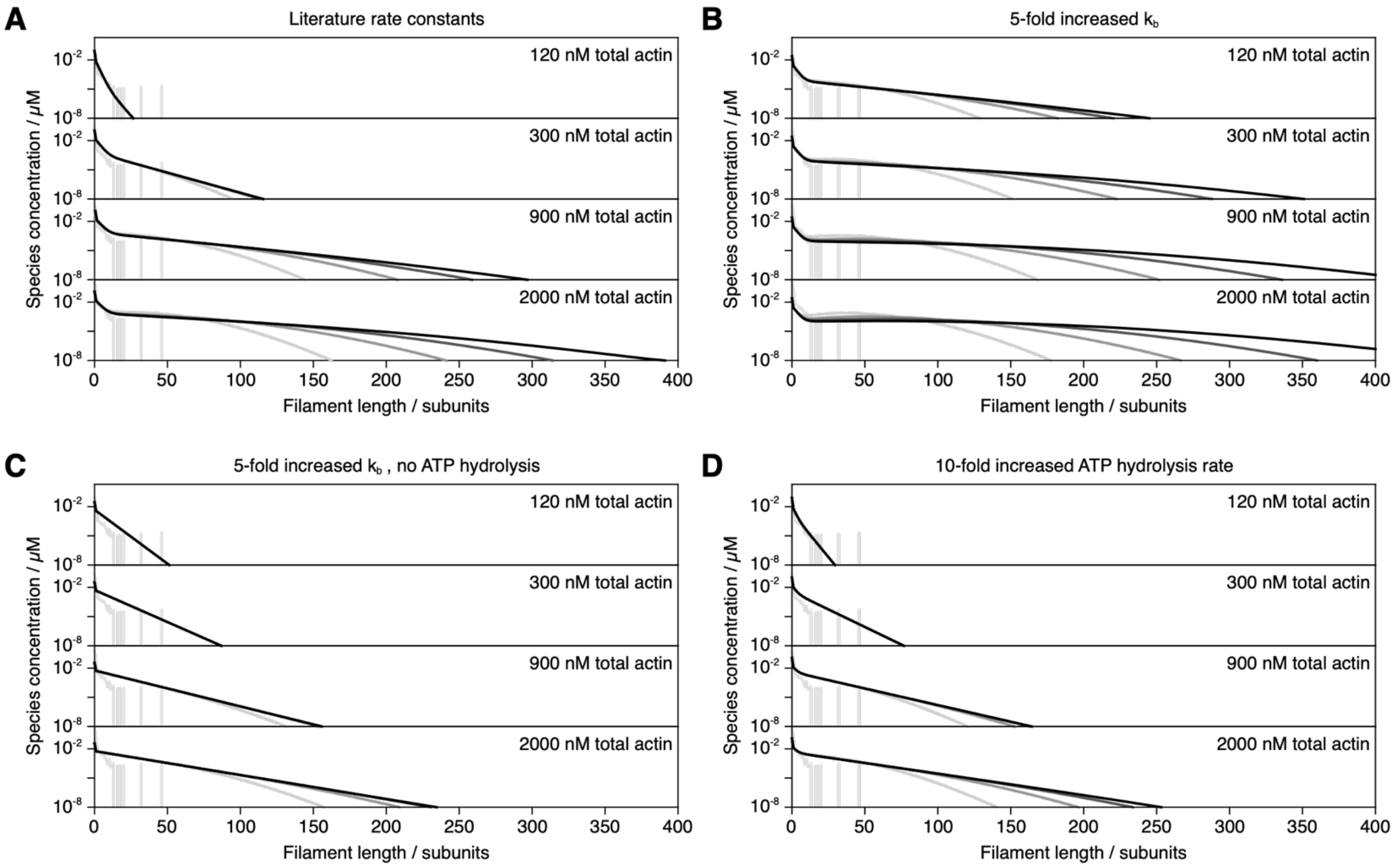

Figure S16 – Potential regulatory switches for actin filament formation

(A) Simulated filament length distribution as a function time (light grey to black: 0, 5, 15, 30, 60 min of polymerization) and total actin concentration, based on the ATP-hydrolysis model with literature rate constants shown in Table S2A. **(B)** Same simulation as in A, but with a 5-fold increased barbed end forward rate constant k_b (58.0 μM^{-1} s^{-1}). **(C)** Same simulation as in B, but with an ATP hydrolysis rate k_{hydr} set to 0. **(D)** Same simulation as in A, but with a 10-fold increased ATP-hydrolysis rate k_{hydr} (0.1 s^{-1}).

Supplementary Tables

Table S1 – Summary of rate constants used for simulations with kinetic scheme 1. (A) Rate constants and sds parameter used for the Sept/McCammon (SM) model simulations (Fig. 2A-D, light blue). **(B)** Rate constants and sds parameter used for the adjusted SM model simulations (Fig. 2A-D, dark blue). Binding Gibbs free energies have been calculated assuming a temperature of 22 °C.

**(A) Sept/McCammon model**	**Ref.**	**Binding equilibrium constant K_{eq} / M^{-1}**	**Binding Gibbs free energy ΔG_{eq} / kJ mol^{-1}**	**(B) adjusted SM model**	**Ref.**	**Binding equilibrium constant K_{eq} / M^{-1}**	**Binding Gibbs free energy ΔG_{eq} / kJ mol^{-1}**
k_1 = 35.7 μM^{-1} s^{-1}<br>k_{-1} = 1.63×10^8 s^{-1}	(*35*)<br>(*35*)	0.22	+3,7	k_1 = 4.2 μM^{-1} s^{-1}<br>k_{-1} = 8.9 s^{-1}		4.72×10^5	-32.1
k_2 = 2.18 μM^{-1} s^{-1}<br>k_{-2} = 1.3×10^3 s^{-1}	(*35*)<br>(*35*)	1.68×10^3	-18.2	k_2 = 12.7 μM^{-1} s^{-1}<br>k_{-2} = 6.6 s^{-1}		1.92×10^6	-35.5
k_3 = 11.1 μM^{-1} s^{-1}<br>k_{-3} = 1.51 s^{-1}	(*35*)<br>(*35*)	7.35×10^6	-38.8	k_3 = 12.05 μM^{-1} s^{-1}<br>k_{-3} = 5.4 s^{-1}		2.23×10^6	-35.9
k_4 = 11.6 μM^{-1} s^{-1}<br>k_{-4} = 1.4 s^{-1}	(*35*, *40*)<br>(*35*, *40*)	8.29×10^6	-39.1	k_4 = 11.6 μM^{-1} s^{-1}<br>k_{-4} = 4.0 s^{-1}	(*40*)	2.90×10^6	-36.5
Bulk scattering smallest detectable species: 140mer				Bulk scattering smallest detectable species: 95mer			

Kinetic scheme 1:

$$A_1 \underset{k_{-1}}{\overset{k_1}{\rightleftharpoons}} A_2 \underset{k_{-2}}{\overset{k_2}{\rightleftharpoons}} A_3 \underset{k_{-3}}{\overset{k_3}{\rightleftharpoons}} A_4 \underset{k_{-4}}{\overset{k_4}{\rightleftharpoons}} \dots \underset{k_{-4}}{\overset{k_4}{\rightleftharpoons}} A_i \underset{k_{-4}}{\overset{k_4}{\rightleftharpoons}} \dots \underset{k_{-4}}{\overset{k_4}{\rightleftharpoons}} A_n$$

Table S2 – Summary of rate constants used for simulations with kinetic scheme 2. (A) Rate constants and sds parameter used for the ATP-hydrolysis model simulations (Fig. 2E-H, 3A, green). **(B)** Rate constants and sds parameter used for the cation exchange model simulations (Fig. 3A-D, red). Binding Gibbs free energies have been calculated assuming a temperature of 22 °C.

**(A) ATP hydrolysis model**	**Ref.**	**Binding equilibrium constant K_{eq} / M^{-1}**	**Binding Gibbs free energy ΔG_{eq} / kJ mol^{-1}**	**(B) Cation exchange model**	**Ref.**	**Binding equilibrium constant K_{eq} / M^{-1}**	**Binding Gibbs free energy ΔG_{eq} / kJ mol^{-1}**
k_1 = 12.9 μM^{-1} s^{-1}<br>k_{-1} = 12.9 s^{-1}	(*40*)<br>(*40*)	1.00×10^6	-33.9	k_1 = 10.6 μM^{-1} s^{-1}<br>k_{-1} = 14.0 s^{-1}	(*31*)	7.58×10^5	-33.2
k_2 = 12.9 μM^{-1} s^{-1}<br>k_{-2} = 2.2 s^{-1}	(*40*)<br>(*40*)	5.86×10^6	-38.2	k_2 = 10.6 μM^{-1} s^{-1}<br>k_{-2} = 5.0 s^{-1}	(*31*)<br>(*31*)	2.12×10^6	-35.7
k_3 = 12.9 μM^{-1} s^{-1}<br>k_{-3} = 2.2 s^{-1}	(*40*)<br>(*40*)	5.86×10^6	-38.2	k_3 = 10.6 μM^{-1} s^{-1}<br>k_{-3} = 5.0 s^{-1}	(*31*)<br>(*31*)	2.12×10^6	-35.7
k_4 = 11.6 μM^{-1} s^{-1}<br>k_{-4} = 1.4 s^{-1}	(*40*)<br>(*40*)	8.29×10^6	-39.1	k_4 = 12.9 μM^{-1} s^{-1}<br>k_{-4} = 2.2 s^{-1}	(*40*)<br>(*40*)	5.86×10^6	-38.2
k_{trans} = 0.01 s^{-1}	(*44*)			k_{trans} = 0.025 s^{-1}	(*31*)		
Bulk scattering smallest detectable species:<br><br>95mer (Horiba fluorometer, Fig. 2F)<br><br>117mer (Varian fluorometer, Fig. 3A)				Bulk scattering smallest detectable species:<br><br>117mer (Varian fluorometer, Fig. 3A)			

Kinetic scheme 2:

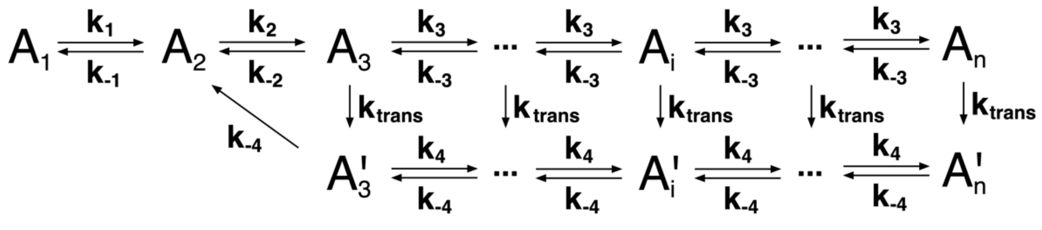

Table S3 – Best-fit rate constants based on Markov-Chain Monte Carlo analysis for the three different actin nucleation models. Rate constants are reported as the median value of the posterior probability distribution function obtained from the MCMC process, with quoted 1σ errors. The rate constants in B are indexed as shown in kinetic scheme 2 in the methods section *Simulation of actin assembly*.

(A) MCMC-fit Sept/McCammon model	(B) MCMC-fit ATP-hydrolysis model
k_1 = 114.13 ± 140.24 μM s^{-1}	k_1 = 15.83 ± 1.27 μM s^{-1}
k_{-1} = 7.76 ± 9.42x10^8 s^{-1}	k_{-1} = 14.37 ± 1.13 s^{-1}
k_2 = 9.50 ± 9.66 μM s^{-1}	k_2 = 18.92 ± 1.36 μM s^{-1}
k_{-2} = 6168.55 ± 6122.27 s^{-1}	k_{-2} = 3.12 ± 0.11 s^{-1}
k_3 = 55.23 ± 26.87 μM s^{-1}	k_3 = 14.72 ± 1.21 μM s^{-1}
k_{-3} = 0.63 ± 0.20 s^{-1}	k_{-3} = 2.38 ± 0.12 s^{-1}
k_4 = 40.66 ± 29.62 μM s^{-1}	k_4 = 12.25 ± 1.27 μM s^{-1}
k_{-4} = 1.40 ± 0.10 s^{-1}	k_{-4} = 1.42 ± 0.01 s^{-1}
AIC = 247,152.1	k_{trans} = 0.0044 ± 0.0001 s^{-1}
BIC = 247,211.8	AIC = 167,954.8
	BIC = 168,022.1
